# Proliferation- and cytotoxic immune signatures identify chemotherapy-responsive bladder tumors in a molecular subtype dependent manner

**DOI:** 10.1038/s41698-026-01588-7

**Published:** 2026-07-03

**Authors:** Aymeric Zadoroznyj, Pontus Eriksson, Carina Bernardo, Lena Tran, Carl-Adam Mattsson, Thomas W. Flaig, Roland Seiler, Catherine M. Tangen, Peter C. Black, Seth P. Lerner, David J. McConkey, Mattias Höglund, Fredrik Liedberg, Gottfrid Sjödahl

**Affiliations:** 1 Department of Translational Medicine, Lund University, Malmö, Sweden; 2 Department of Urology, Skåne University Hospital, Malmö, Sweden; 3 Division of Oncology, Department of clinical sciences, Lund University, Lund, Sweden; 4 Division of Medical Oncology, University of Colorado School of Medicine, Aurora, CO USA; 5 Department for BioMedical Research, Urology Research Laboratory, University of Bern, Bern, Switzerland; Department of Urology, Hospital Center Biel, Spitalzentrum Biel, Biel, Switzerland; 6 SWOG Statistical Center, Fred Hutchinson Cancer Research Center, Seattle, WA USA; 7 University of British Columbia, Vancouver, BC Canada; 8 Scott Department of Urology, Dan L. Duncan Cancer Center, Baylor College of Medicine, Houston, TX USA; 9 Department of Urology, University of Rochester Medical Center, Rochester, NY USA

**Keywords:** Biomarkers, Cancer, Computational biology and bioinformatics, Oncology

## Abstract

Neoadjuvant chemotherapy (NAC) followed by radical cystectomy (RC) has been the standard care for muscle-invasive bladder cancer (MIBC) for two decades. One third of NAC-treated patients achieve pathologic complete response (pT0N0), a proxy for improved survival after RC. Predicting response already at transurethral resection of bladder tumor (TUR-BT) would enable selective use of NAC, minimizing exposure to ineffective therapy. We aimed to identify tumor mRNAs associated with response across multiple transcriptomic studies, prioritizing subsequent biomarkers for validation. Three NAC-treated cohort with tumor transcriptomic profiles were included. Differential mRNA-expression analysis and subtype classification according to the Lund Taxonomy were performed. Within each cohort and subtype, genes were ranked by differential expression, and integrated into a meta rank-score. Survival associations of top genes in the NAC-cohorts were used to select candidate biomarkers for protein validation. Proliferation/late cell-cycle gene predicted response in Urothelial-like subtype and cytotoxic T- and NK-cell-related genes predicted response in Basal/Squamous tumors. These findings were validated by immunostainings for CCNB1 and NKG7, respectively. This integrative framework suggests a complex picture in which two NAC-predictive signals identify responders in a subtype dependent manner. The framework can be updated as new datasets become available, providing dynamic exploration of NAC-predictive biomarkers in MIBC.

## Introduction

Cisplatin-based neoadjuvant chemotherapy (NAC) prior to radical cystectomy (RC) provides a modest but significant survival benefit of 5–8% for patients with muscle-invasive bladder cancer (MIBC)^1,2^. The most commonly used NAC regimens are dose-dense MVAC (methotrexate, vinblastine, doxorubicin and cisplatin) and GC (gemcitabine and cisplatin), which may be combined with immune checkpoint inhibition^3^, or in the near future after EMA-approval exchanged by pre- and postoperative enfortumab vedotin plus pembrolizumab^4^. Although the benefit of NAC is mediated by treating micrometastatic disease, pathologic complete response (pCR) in the bladder and excised lymph-nodes (pT0N0) is strongly associated with favorable long-term survival^5–7^. In non-responders on the other hand, outcomes might even worsen due to NAC by further delaying RC^8,9^. This underscores the need for predictive biomarkers to identify patients most likely to respond, and reserve NAC for those patients.

Several tumor features have been associated with NAC response, including molecular subtypes^10–12^, genomic alterations in DNA damage response genes such as ERCC2 and BRCA2^10,13^, immune infiltration^14–16^, and histological architecture^17^. However, validation of these biomarkers and understanding of their relationship with subtype classification is still unclear^18,19^, and no molecular predictors are currently used for clinical decision-making. We hypothesize that two main shortcomings have limited progress in this field. First, MIBC has until now been treated as one disease, without considering that molecular subtypes may have different response and resistance mechanisms. Second, the reliance on single cohorts with small sample sizes lead to high false discovery rate (FDR) and low concordance between study results.

Here, we combine the three largest transcriptomic datasets with MIBC patients treated with NAC, to explore gene expression patterns and candidate biomarkers predictive of pCR within each molecular subtype.

## Results

### Response to NAC is impacted by tumor molecular subtype

**Fig. 1:**
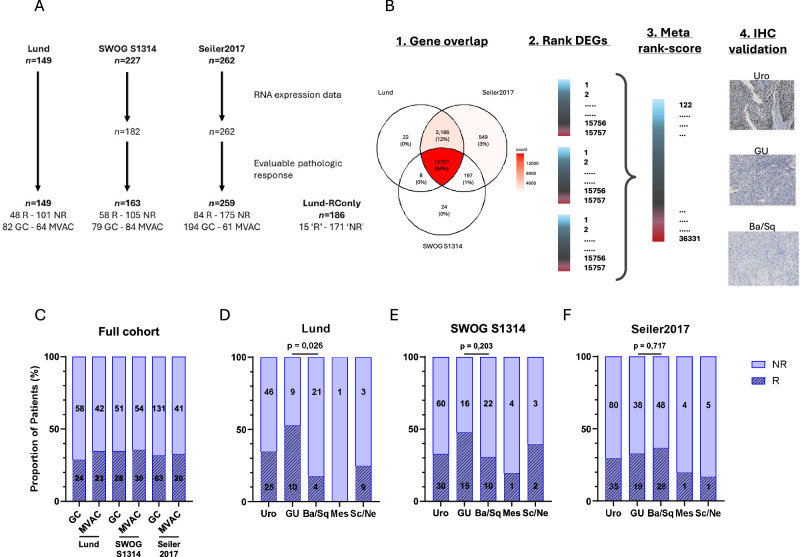
Classification of bladder cancer by subtype highlights discrepancies in response to NAC. **A** Schematic representation of the three NAC-treated cohorts and patient inclusion criteria. **B** Description of the research pipeline. Only genes common to all cohorts were selected. Limma differential expression analysis was performed on the full cohort and by molecular subtypes, and the results were combined to obtain a meta rank-score. The most promising biomarkers identified using the meta rank-score were selected and validated by immunohistochemistry. Comparison of treatment response between the full cohort (**C**) and among the 5 molecular subtypes within each cohort: Lund (**D**), SWOG S1314 (**E**) and Seiler2017 (**F**). R responder defined as pCR, NR non-responder.

We analyzed RNA expression data and pathologic response outcomes from three bladder cancer cohorts treated with MVAC or GC; the “Lund” (*n* = 149), the “SWOG S1314” (*n* = 163) and the “Seiler2017” (*n* = 259) cohorts (Fig. 1A). In each cohort, patients with pCR were compared with patients who had residual disease in the cystectomy specimen. An additional “RC-only” cohort (*n* = 186), composed of patients who underwent RC without NAC, was used as a control. A schematic overview of the analytical pipeline is shown in Fig. 1B.

Each patient cohort was classified into five main molecular subtypes according to the Lund Taxonomy, where the Uro subtype can be further divided into three minor subtypes (UroA, B and C) (Figs. S1–3). Treatment response, defined as pCR (ypT0N0), was stratified by NAC-regimen (GC and MVAC) (Fig. 1C) and by molecular subtype within each cohort (Fig. 1D–F). Subtype specific differences were most evident in the Lund and SWOG S1314 cohorts, where patients with the GU subtype responded better than those with a Ba/Sq subtype (*p* = 0.026 and *p* = 0.203, respectively) (Fig. 1D, E). This pattern was not observed in the Seiler2017 cohort (*p* = 0.717) (Fig. 1F). The most prevalent Uro subtype had a consistent intermediate response rate across all cohorts, while the less prevalent Mes and Sc/Ne subtypes were too infrequent for analysis.

### Meta rank-score analysis highlights subtype-dependent biomarkers of NAC sensitivity

**Fig. 2:**
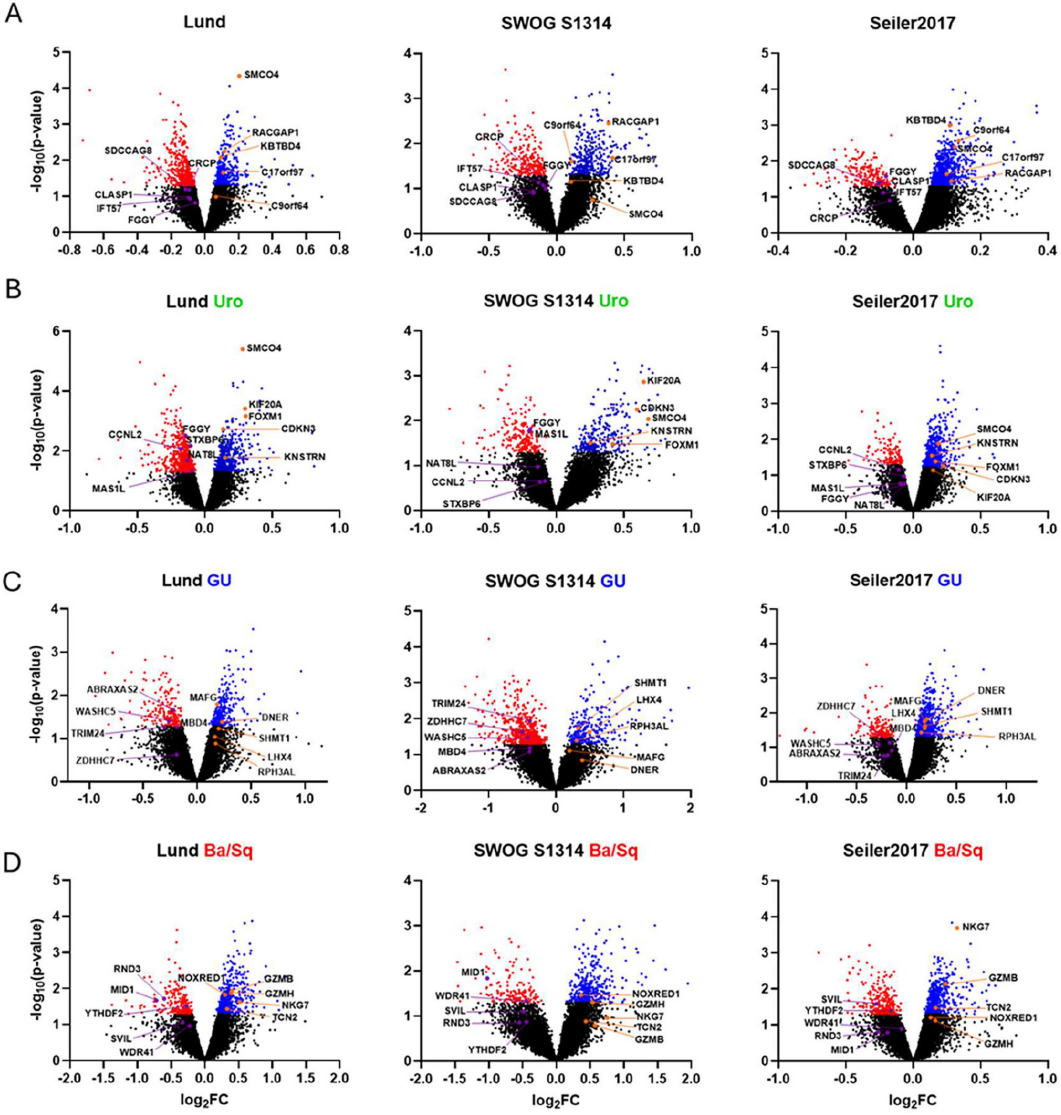
Differential expression analysis by treatment response in each cohort and in three molecular subtypes. Volcano plots representing the differential expression analyses performed on the NAC-treated full cohort (**A**) and the three major molecular subtypes: Uro (**B**), GU (**C**) and Ba/Sq (**D**). Genes with statistical significance (*p* ≤ 0.05) are marked in red for downregulation and blue for upregulation, and the 5 genes with the highest and lowest meta rank-score in each volcano plot are highlighted.

Differential expression analysis by pCR status in each cohort identified 817, 566, and 1084 differentially expressed genes (DEGs) in the Lund, SWOG S1314 and Seiler2017 cohorts, respectively (Fig. 2A). The same analysis was performed within each molecular subtype for all NAC cohorts (Fig. 2B–D) and for MVAC versus GC regimen (Fig. S4). Similar analyses were performed per subtype within the Lund RC-only control cohort (Fig. S5). Genes consistently detected across the three datasets were ranked according to their association with pCR, yielding a weighted meta rank-score. Lower meta rank-scores corresponded to genes positively associated with response, whereas higher scores indicated association with non-response. Meta rank-scores for all 15,757 genes are presented in Data S1. The approach identified *KBTBD4*, *C17orf97*, *RACGAP1*, *C9orf64* and *SMCO4* as genes most associated with NAC sensitivity in the full cohorts, with subtype-specific top genes including *SMCO4*, *FOXM1*, *KNSTRN*, *CDKN3* and *KIF20A* in Uro; *MAFG*, *SHMT1*, *LHX4*, *DNER* and *RPH3AL* in GU; and *NKG7*, *NOXRED*, *GZMB*, *GZMH* and *TCN2* in Ba/Sq (Table 1). Two clear patterns emerged from the analysis: proliferation genes (e.g., *FOXM1, CDKN3, and KIF20A*) were associated with NAC sensitivity in the Uro subtype, whereas genes expressed by cytotoxic immune cells (e.g., *GZMB*, *GZMH* and *NKG7*) predicted a favorable response in the Ba/Sq subtype. In contrast, the top genes associated with non-response did not reveal any consistent biological theme in any of the subtypes. Across all analyses, the lowest meta rank-score was observed in the Uro subtype for the gene *SMCO4* with ranks 1, 49, and 96 in the three cohorts. Conversely, the highest meta rank-score was obtained in the Ba/Sq subtype for *SVIL*, which encodes the actin regulatory protein supervillin^20^. The top genes associated with response and non-response in each subtype are highlighted in the corresponding volcano plots for each NAC cohort (Fig. 2A–D). Taken together, comparison of the meta rank-scores in the full cohort and the subtype-informed analyses showed increased biological specificity when accounting for molecular subtypes. Consequently, in the full cohort, the proliferation and immune-cytotoxicity signals identified in Uro and Ba/Sq subtypes, respectively, were diluted and masked by mixed signatures and other genes. Importantly, the top-ranked genes in each subtype showed no association with pCR in the Lund RC-only cohort (i.e., pT0N0 after transurethral resection of bladder tumor (TUR-BT) alone), suggesting that these signals reflect NAC response rather than identifying patients with low disease burden (Table 1 and Fig. S5).

**Table 1:** Top 5 genes with the highest and lowest meta rank-score in the full cohort and in each major subtype

Subset	Direction	Top 5 Genes	Rank Lund	Rank SWOG S1314	Rank Seiler2017	Meta rank-score	Rank Lund RC-only
Full cohorts	Response	*KBTBD4*	43	494	13	**303.4**	12,735
		*C17orf97*	118	149	334	**500.7**	12,889
		*RACGAP1*	32	19	519	**553**	1871
		*C9orf64*	672	183	28	**630**	1873
		*SMCO4*	1	1291	48	**722.8**	9423
	Non- response	*FGGY*	14,515	15,342	15,674	**34,624.5**	12,344
		*CLASP1*	14,820	15,177	15,602	**34,696.2**	9568
		*CRCP*	15,256	15,262	15,235	**34,702.2**	1826
		*IFT57*	14,878	15,402	15,467	**34,722.4**	14,714
		*SDCCAG8*	15,272	15,119	15,628	**35,032.6**	5971
Uro	Response	*SMCO4*	1	49	96	**122.3**	5363
		*FOXM1*	12	172	307	**405.9**	8001
		*KNSTRN*	175	156	199	**412.4**	8219
		*CDKN3*	26	32	417	**453.3**	6850
		*KIF20A*	9	9	574	**585.5**	2801
	Non- response	*NAT8L*	15,527	15,243	15,154	**34,815.5**	12,781
		*STXBP6*	15,672	14,457	15,459	**34,819.4**	14,288
		*MAS1L*	15,261	15,701	15,178	**34,878.2**	8625
		*CCNL2*	15,655	14,369	15,597	**34,898.7**	1231
		*FGGY*	15,717	15,707	15,164	**35,211.0**	10,312
GU	Response	*MAFG*	60	444	182	**459.1**	7222
		*SHMT1*	278	106	259	**523.9**	8668
		*LHX4*	484	136	299	**734.8**	8167
		*DNER*	182	852	154	**736.0**	10,106
		*RPH3AL*	755	234	413	**1104.2**	10,916
	Non- response	*ZDHHC7*	14,455	15,527	15,508	**34,509.9**	1652
		*ABRAXAS2*	15,694	14,898	15,071	**34,678.3**	7297
		*MBD4*	15,566	15,079	15,289	**34,894.3**	6844
		*TRIM24*	15,569	15,650	15,035	**34,940.7**	9625
		*WASHC5*	15,619	15,410	15,462	**35,280.1**	2902
Ba/Sq	Response	*NKG7*	179	886	2	**599.5**	8462
		*NOXRED1*	110	229	635	**837.5**	12,770
		*GZMB*	104	1366	49	**840.6**	5930
		*GZMH*	90	342	800	**1046.4**	6250
		*TCN2*	260	1,073	323	**1079.2**	9345
	Non- response	*RND3*	15,712	15,143	14,938	**34,686.8**	1526
		*WDR41*	15,261	15,629	15,091	**34,753.6**	946
		*MID1*	15,707	15,730	14,893	**34,944.5**	6188
		*YTHDF2*	15,669	15,167	15,584	**35,312.9**	3269
		*SVIL*	15,404	15,462	15,653	**35,336.2**	11,540

For each gene, the rank in each cohort and their exact meta rank-score is described. The Lund RC-only rank is shown as a control.

The values in bold indicate the meta rank-score, which are the primary results presented in Table 1.

### Broad proliferation and immune cytotoxicity signatures have strong subtype-dependent impact on NAC-response

**Fig. 3:**
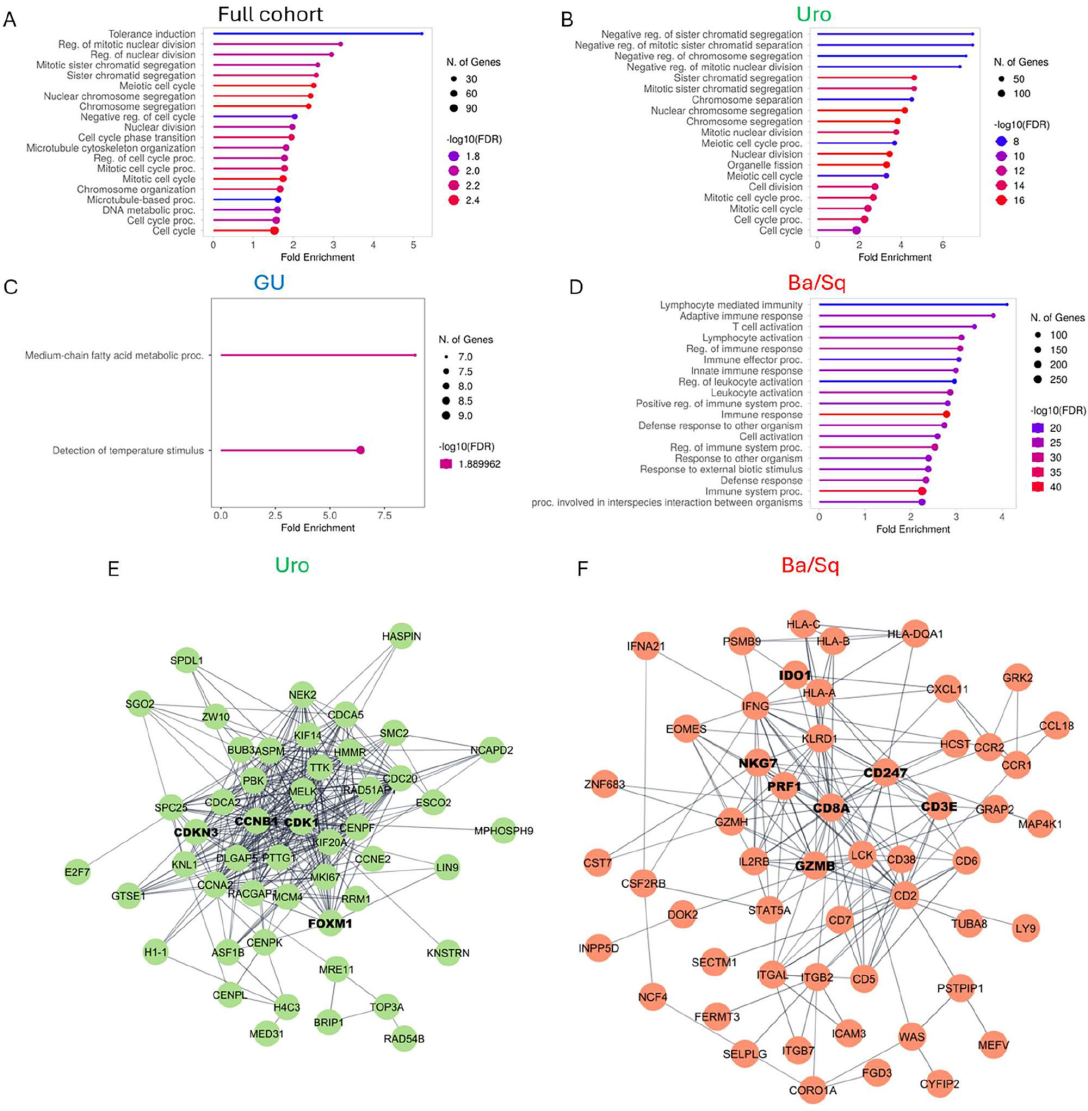
Cellular processes related to treatment response are specific to molecular subtypes. GO term analysis using “biological processes,” from the 5% of genes with the lowest meta rank-score in the full cohort (**A**) and major molecular subtypes: Uro (**B**), GU (**C**) and Ba/Sq (**D**). At most, the top 20 terms with the highest fold enrichment are described. Major protein-protein interaction observed in the top 1% of genes with the lowest meta rank-score in the Uro (**E**) and Ba/Sq (**F**) subtypes using STRING.

To systematically investigate the biological pathways underlying NAC response, we performed GO analysis using the 1% or 5% of genes with the lowest and highest meta rank-score, both for the full cohort and within each major subtype (Uro, GU and Ba/Sq) (Figs. 3A–D and S6A–D). Genes associated with non-response to NAC (highest meta rank-scores) showed no consistent biological patterns, with only modest enrichment for cytoskeleton organization in the full cohort and within the Ba/Sq subtype. However, for genes associated with treatment sensitivity, a strong enrichment of cell cycle and cell proliferation related processes was observed (Fig. 3A). Similar proliferation-related genes were further enriched in the Uro subtype, with approximately doubled levels of enrichment (top 20 terms: ~2–8 fold in the full cohort versus ~4–20 fold in Uro) and markedly stronger FDR adjusted *p*-values (10^−3^–10^−6^ for full cohort versus 10^−8^–10^−12^ for Uro, Fig. 3B). We observed no statistically robust enrichments related to response in the GU subtype (Fig. 3C), whereas the Ba/Sq subtype showed strong enrichment (top 20 terms: ~2–4-fold enrichment and FDR-adjusted *p* = 10^−20^–10^−40^) for immunity- and cytotoxicity-related processes (Fig. 3D). These subtype-specific enrichments were maintained when analyzing the top 1% and the top 5% of genes (Fig. S6). These results were further validated by GSEA analysis: 128 and 147 gene sets were significantly enriched (FDR ≤ 0.05) in the Uro and Ba/Sq subtypes, respectively, corresponding to cell proliferation and cytotoxic immune response (Fig. S7), whereas no significant enrichments were identified in GU. To further investigate response-associated biology, we used STRING to test if any protein interaction networks were differentially regulated depending on the meta rank-score (Fig. 3E, F). Consistent with the ontology analysis, no response-related networks were detected in the GU subtype. As expected, however, the response-associated genes in the Uro subtype formed a dense proliferation-related network with discernible substructures relating to nucleosome and kinetochore structures as well as homologous recombination repair (Fig. 3E). An interactome related to T/NK-cell immunity was also seen for response-associated genes in the Ba/Sq subtype (Fig. 3F), although it was not as dense as the proliferation network in the Uro subtype. The network captured various aspects of the T cell interactome, e.g., T-cell receptor components (*CD3E*, *CD8A*), *IFNG*, cytotoxic granule proteins (*PRF1*, *GZMH*, *NKG7*), and immune checkpoints (*CD247*, *CTLA4*, *IDO1*). In the full cohort, the interactome profile was primarily driven by the Uro subtype, while a more limited overlap was noted with the Ba/Sq subtype (Fig. S8). Similar to the GO analysis, genes associated with non-response did not form coherent networks suggesting that resistance mechanisms may be pleiotropic and less subtype-specific than the positive determinants of NAC sensitivity.

### MIBC Consensus classification identifies the same subtype dependent signatures

Differential expression analysis and meta rank-score calculation were also conducted for each subtype of the MIBC consensus molecular classification and for a combination of LumNS and LumP (corresponding to the LundTax Uro subtype) (Data S2 and Fig. S9). GO analysis was then performed on the top 5% of genes with the lowest meta rank-scores within each group (Fig. S10). Consistent with previous findings, the Ba/Sq subtype showed enrichment of immune- and cytotoxicity-related processes among genes associated with treatment response (Fig. S10A), though with weaker *p*-values than those observed in the LundTax classification (FDR-adjusted *p* = 10⁻²–10⁻⁴). In the LumNS subtype, corresponding to a subset of the LundTax Uro subtype (Fig. S9), response-associated genes were enriched for cell cycle and mitochondrial fission signatures (Fig. S10B), again with weaker *p*-values than in the corresponding LundTax Uro subtype (FDR-adjusted *p* = 10⁻³–10⁻⁹). In LumNS and LumP combined, it is mainly cell cycle and proliferation signatures that are highlighted (Fig. S10C). No statistically robust enrichments were detected for the LumP, LumU, or stroma-rich subtypes. Thus, the consensus classification supports our observations based on the LundTax classification, although with reduced statistical significance.

### Resampling-based analysis and pairwise cohort analysis confirm subtype specific predictive signals

**Fig. 4:**
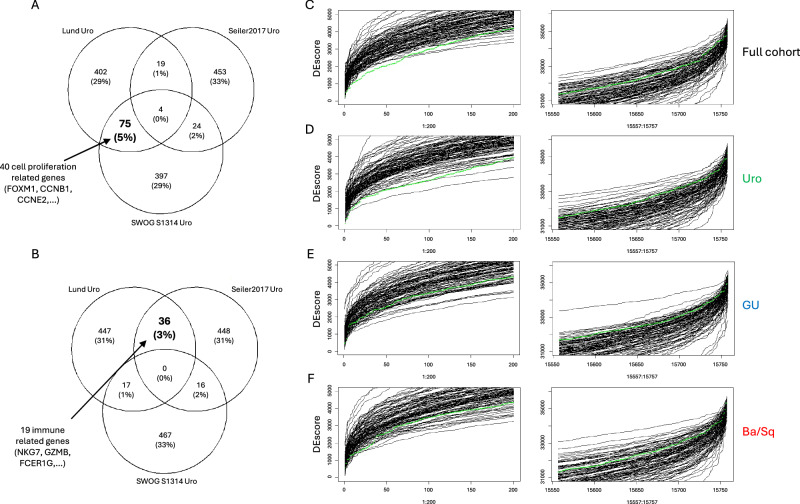
Meta rank-score resampling-based analysis highlights genes associated with treatment sensitivity. Venn diagram showing the number of genes associated with treatment sensitivity (500 genes with the lowest meta rank-score) that are shared across the different cohort in the Uro (**A**) and Ba/Sq (**B**) subtypes. Resampling-based analyses were performed for the full cohort (**C**) and in each major subtype (Uro, **D**; Ba/Sq, **E**; GU **F**). Line plots show meta rank-score distributions across the 200 genes with the lowest (left) and highest (right) scores. The observed meta rank-score distribution is shown in green and distributions from resampled data in black.

Next, we assessed how informative response-related signals in the TUR-BT transcriptome are for predicting NAC response, and whether these signals are consistent across studies. For this purpose, we first investigated pairwise concordance of top DEGs across the three NAC cohorts (Data S3). We found that 79 of the top 500 response-associated genes in the Uro subtype overlapped between the Lund and the SWOG S1314 cohorts, with 40 of the overlapping genes being involved in cell proliferation. In contrast, the Seiler2017 cohort showed poor overlap with both the Lund and SWOG S1314 (23 and 28 genes, respectively) (Fig. 4A). For the top 500 response-associated genes in the Ba/Sq subtype, we observed larger overlap between Lund and Seiler2017 cohorts (36 genes) than for SWOG S1314 with either Lund or Seiler2017 cohorts (17 and 16 genes, respectively) (Fig. 4B). Among these 36 genes, 19 belong to an immune-related GO signature. These observations are supported by GO term analyses performed on the individual cohorts. Enrichment of cell proliferation signatures was observed in the Uro subtype in both the Lund and SWOG S1314 cohorts (Fig. S11A, B), but not in the Seiler2017 cohort (Fig. S11C). Conversely, enrichment of immune-related signatures was observed in the Ba/Sq subtype in the Lund and Seiler2017 cohorts (Fig. S11D, E), however not in the SWOG S1314 cohort. Next, we identified the response associated genes stratified by chemotherapy regimen (GC vs. MVAC). These data support a link between proliferation and treatment response in the Uro subtype that is more pronounced among patients that received MVAC compared to GC (Figs. S12 and S13).

To test how many more pCR-associated genes our approach might identify beyond false positive findings, we then applied a resampling-based approach, calculating 100 permutations of the meta rank-score from the same three datasets, but with response labels randomly assigned. The extremes of these null distributions are plotted in Fig. 4C–F. In the full cohort, the top 150 response-associated genes obtained more extreme meta rank-scores than 95% of the permuted distributions (Fig. 4C). Subtype-specific analyses revealed that in the Uro subtype, more than 200 genes were more strongly associated with NAC response than expected by chance (Fig. 4D). In contrast, the GU subtype showed no significant deviations from the null distribution, even among the genes with the lowest score (Fig. 4E). Finally, in the Ba/Sq subtype, approximately 40 genes obtained more extreme scores than expected (Fig. 4F). Notably, no significant differences were detected between the real and permuted meta rank-score distributions in the non-response direction in any cohort.

In summary, these results confirm that substantial predictive signals for NAC sensitivity are present in MIBC expression profiles, which can be attributed mainly to a few hundred genes in the Uro subtype and several dozens of genes in the Ba/Sq subtype. The lack of robust signals in the GU subtype, and in the direction of non-response, indicates that few, if any, genes are predictive of NAC response in these settings.

### Protein-level validation of candidate subtype-dependent biomarkers

**Fig. 5:**
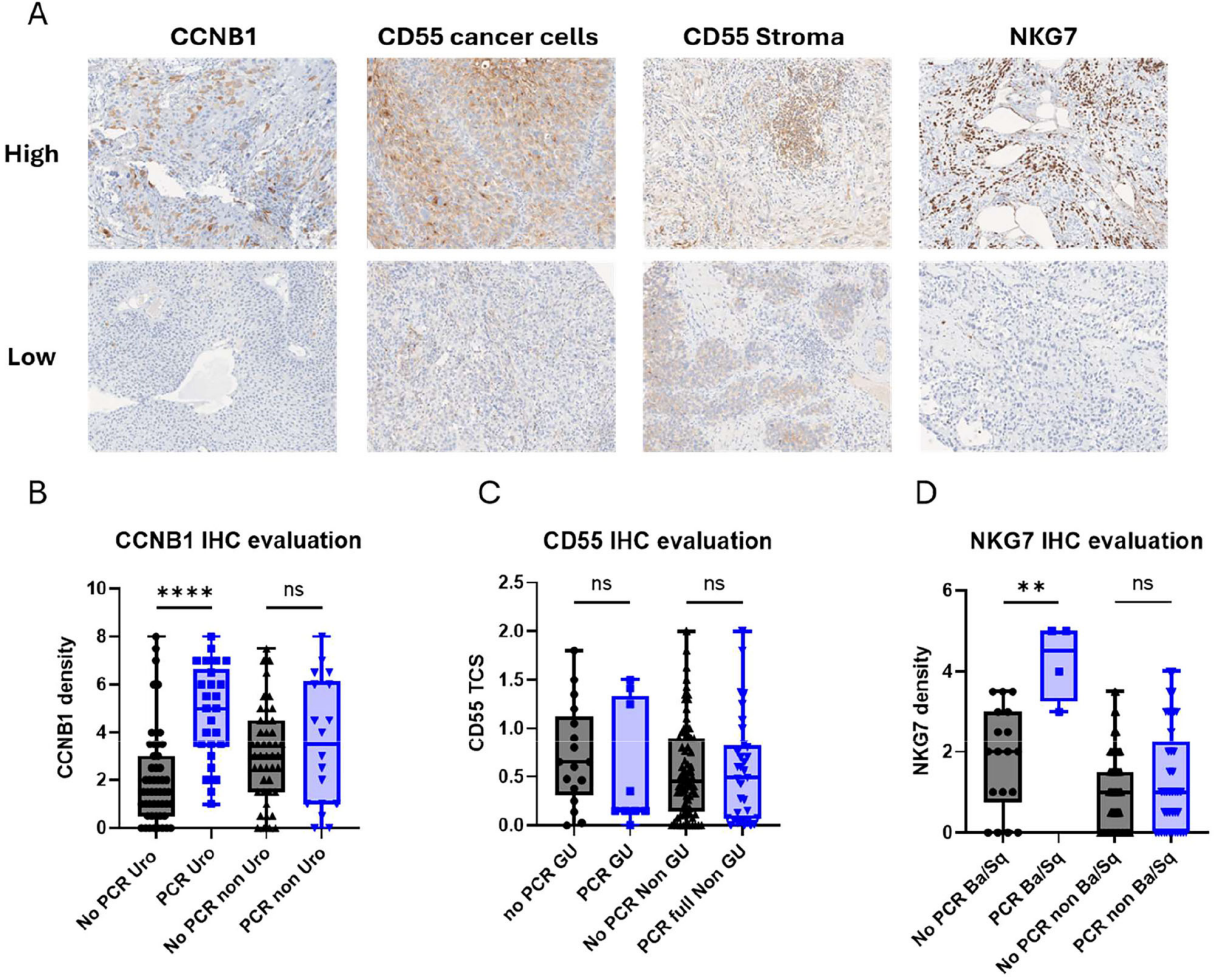
IHC evaluation of candidate biomarkers confirms their specific subtype effect on treatment sensitivity. **A** The upper panels show strong protein staining in a TMA core from the Lund cohort, with high expression observed in tumor cells for all biomarkers and in the stroma for CD55. The lower panels show weak protein expression in the same TMA core. **B**–**D** Protein expression was evaluated in cancer cells for CCNB1 and NKG7 and in both cancer cell and stroma compartments for CD55. Expression levels were then compared across tumor subtypes stratified by treatment response: Uro and non-Uro for CCNB1 (**B**), GU and non-GU for CD55 (**C**), and Ba/Sq and non-Ba/Sq for NKG7 (**D**).

Subsequently, we used the meta rank-scores to prioritize six candidate biomarkers for protein-level validation. In addition to an extreme meta rank-score in the NAC cohorts and not in the RC-only cohort, these biomarker candidates should have a commercially available antibody suitable for IHC and show a significant univariate association to cancer-specific survival in the Lund NAC cohort for which tumor tissue embedded in TMAs was available. Based on these criteria, we selected *SMCO4* and *CCNB1* (response in Uro), *CD55* (non-response in GU), *NKG7* (response in Ba/Sq), *ALDH1A1* (response in Uro and non-response in GU), and *CYP4Z1* (non-response in Uro) (Data S1). Adequate staining was achieved for four of the six candidate markers: CCNB1, CD55, NKG7, and ALDH1A1. IHC-scores correlated positively with corresponding gene expression values (Pearson *r* = 0.47-0.52) (Fig. S14). IHC-scores stratified by response-status were then analyzed in the intended subsets. The density of CCNB1-positive cancer cells was associated with NAC response in the Uro subtype only (*p* ≤ 0.0001) (Fig. 5B). This association was not related to the chemotherapy regimen (Fig. S15). CD55, which was expressed in both cancer and non-cancer cells, showed a non-significant trend toward non-response in the GU subtype, with no effect in the other subtypes (Fig. 5C). No association with NAC response was observed for ALDH1A1 staining across molecular subtype or cellular compartment (cancer cells, non-cancer cells generally, or in stellate-shaped myeloid cells) (Fig. S16). Finally, despite few responders in the Ba/Sq subtype, the density of NKG7-positive immune cells was significantly associated with NAC response in this subset (*p* = 0.003) (Fig. 5D). In the Lund RC-only cohort, no association with pCR was observed for CCNB1 or NKG7. For CD55, a moderate association was observed, but only in non-GU tumors and in the opposite direction of that seen in the NAC cohorts. A weak association with pCR status was also seen for ALDH1A1 in the Uro subtype (Fig. S17).

A subtype-specific assessment of CCNB1 and NKG7 immunostaining as predictive biomarkers in the training data is shown in (Fig. S18). CCNB1 and NKG7 resulted in pCR-prediction with an AUC of 0.831 and 0.944 in the intended subtypes. At the optimal cut-point this resulted in sensitivity of 0.77 and 0.75, specificity of 0.76 and 1.0, and predictive accuracy of 0.77 and 0.95 Given the response and survival outcomes of the Lund-cohort training data, a decision curve analysis (Fig. S19) indicated that treating according to biomarker status would result in a clinical net benefit only for patients in the intended subtypes (Uro for CCNB1 and Ba/Sq for NKG7). In summary, IHC staining of CCNB1 and NKG7 protein expression captures the chemotherapy sensitivity associated with proliferation in the Uro subtype, and cytotoxic immune activity in the Ba/Sq subtypes, respectively.

## Discussion

The major finding of this work is that broad biological programs relating to proliferation and to cytotoxic T- and NK-cells are strongly associated with pathologic response, but in a subtype-dependent manner. Proliferation genes were the strongest predictors of response in the Urothelial-like (Uro) subtype, whereas cytotoxic immune signatures were predictive specifically in the Basal/Squamous (Ba/Sq) subtype. Notably, previous studies have reported positive associations between neoadjuvant therapy response and various measures of proliferation^17,21,22^ as well as T-cell activity^10,14,15,23,24^, but these findings were not previously linked to a specific molecular subtype. Galsky and colleagues showed in the IMvigor130 trial that pre-existing adaptive immunity, including T- and NK-effector signatures, was required for the improved effect of cisplatin over carboplatin^24^. Although the clinical setting with palliative treatment for locally advanced and metastatic disease is different from the current neoadjuvant context, this work highlights the role of cisplatin in enhancing tumor immunogenicity and promoting adaptive anti-tumor immunity. Similarly, Groeneveld et al., using data from the VESPER-trial, reported that tumors with mixed molecular subtype and low proliferation had poor pCR rate^12^. It remains to be seen if this is in line with our results, since tumors with a mixed subtype often involved the Stroma-rich MIBC-consensus subtype, which has a lower proliferation signal due to higher stromal content in bulk tissue samples. The LundTax classification avoids conflation of tissue sample purity with molecular subtype, and it would be interesting to apply proliferation and T- and NK-cell signatures in the light of LundTax classification to the IMvigor130 and VESPER datasets.

A major limitation in the search for predictive biomarkers of NAC response in MIBC is that no transcriptomic studies have randomized patients to NAC versus RC-only. Therefore, current evidence is limited to single-arm clinical trials and retrospective cohort studies, all hampered by difficulties to separate predictive effect from general prognostic associations. In this context, pCR is an important surrogate endpoint because survival after RC depends on several factors other than treatment response e.g., clinical T- and N-stage, and presence of lymphovascular invasion or a high-risk variant histology^25,26^. However, the probability of obtaining a pathologic response in the bladder (pT0) does not only depend on the tumor’s intrinsic treatment sensitivity, but also on the initial disease burden and TUR-BT completeness. The contribution of TUR-BT to pCR rates in cT2 disease has been estimated at 38%, with pCR observed in 28% of patients receiving NAC compared to 9% without^27^. In non-organ confined diseases, pCR rates are lower but NAC continues to confer a survival benefit^28^. Furthermore, differences in treatment regimens (GC or MVAC); inclusion of clinically node-positive patients despite being a separate induction indication for preoperative chemotherapy compared to NAC; the number of NAC cycles; response definitions (pT0N0 versus downstaging to pTis/Ta/T1); population-based versus selected cohorts; and diverse patient populations are examples of confounders that likely affect studies without a control arm not receiving NAC. Such variability may explain the low reproducibility of biomarkers reported in individual studies. In this work, we hypothesize that genes robustly associated with pathologic response should demonstrate consistent signals across multiple studies, even if they are not the top DEGs in each individual study. Furthermore, since MIBC molecular subtypes represent biologically distinct entities^29,30^, we propose that different subtypes may respond or fail to respond to NAC through distinct biological mechanisms and that subtype-specific predictive genes may be obscured in pan-MIBC investigations.

To mitigate these limitations, we integrated all available NAC-treated cohorts with transcriptomic data and sufficient sample size. By relying on gene ranking rather than absolute expression values, we minimize cohort-specific biases. We also applied uniform response definitions and inclusion criteria regarding number of received NAC cycles. Furthermore, by stratifying the analysis according to the cancer cells’ intrinsic phenotypic state with the LundTax classifier^31^, we accounted for the biological heterogeneity of MIBC and could dissect NAC response biology in a subtype-informed manner. Thus, small scale studies, although underpowered for robust conclusions, can still be contextualized in light of our meta rank-scores. As an example, Murphy et al. reported DEGs in a single-center MIBC cohort (*n* = 18), highlighting expression of *CRKL* in responders and *SPRY1* in non-responders^32^. Of these, only *SPRY1* showed a consistent non-response association in the Uro (rank 178) and Ba/Sq subsets, but surprisingly the opposite association in GU (rank 205 response-associated). Given that only two clear GU/LumU tumors were included in their discovery cohort, subtype imbalance is a possible explanation for this discrepancy^32^. Thus, our result nuanced the interpretation to suggest that *SPRY1* could be a candidate subtype-dependent NAC biomarker warranting further investigation.

We selected six candidate markers to validate by IHC, of which two did not yield satisfactory staining (SMCO4 and CYP4Z1), one failed to reproduce the expected association (ALDH1A1), and one showed a non-significant trend consistent with the mRNA data (CD55). Statistically significant associations were confirmed for proliferation (CCNB1, Uro) and cytotoxic immunity (NKG7, Ba/Sq), both with effect sizes indicating potential clinical utility in the Lund NAC cohort. Although not an independent cohort validation, these findings show that our RNA-level observations reflect true biological processes, namely the density of proliferating cancer cells and intra-tumoral cytotoxic lymphocytes, rather than tissue sample purity effects or technical artifacts. These biomarkers showed promising statistical metrics in the training data. Furthermore, decision curve analysis indicated that a biomarker-informed approach would result in a clinical net benefit compared to the situation where every eligible patient with a Uro or Ba/Sq tumor is treated. Whether these results translate into real clinical utility remains to be shown in external prospective validation and subsequent clinical studies.

The main limitation of our work is its restriction to expressed biomarkers at the bulk transcriptomic level. Some determinants of NAC response may only become evident through temporally or spatially resolved data, by accounting for non-linear relationship between genes, or through integration with proteomic and genomic information. In this sense, our work captures only part of the context dependency of NAC response prediction. Nevertheless, central aspects of MIBC biology including molecular subtype, immune-infiltration and proliferation are well captured in the bulk transcriptome, and our results suggest that their predictive potential is now beginning to emerge. Another limitation is that our analysis was based on microarray data, whereas RNA-sequencing, the current technical standard, was not used for the suitable NAC cohorts. Still, by relying on internal gene-ranks within each cohort, our approach is inherently robust across platforms, meaning that as additional RNA-seq cohorts become available, they can be added to refine and expand the subtype-specific meta rank-scores. To bring additional value and bridge the interface to clinical implementation, the current subtype-informed predictive information could be applied in ex-vivo models on patients’ tumors^33^ to better identify responders to standard of care and alternative therapies.

In summary, we present the first subtype-informed, integrated analysis of NAC-response biomarkers across the largest publicly available transcriptomic cohorts. Our findings show that proliferation and cytotoxic T- and NK-cell signatures are predictive of NAC-response specifically in the Uro and Ba/Sq subtypes, respectively. The resulting meta rank-score tables (Data S1) provide a resource for biomarker discovery, which can be further strengthened as new transcriptomic data becomes available.

## Methods

### Identification and acquisition of datasets

The literature was screened to identify cohorts of patients with MIBC receiving cisplatin-based NAC followed by RC. Cohort inclusion criteria were as follows; transcriptomic data for at least 100 patients to enable subtype-specific analysis, available response status (ypTN) following at least 2 cycles of NAC. By December 2024, three patient cohorts were selected: one in-house dataset, the “Lund” Chemo-cohort^11^ (*n* = 149), and two external datasets, the “SWOG S1314” cohort (*n* = 227)^34,35^ and the “Seiler2017” cohort (*n* = 262)^36^. Within the SWOG S1314 cohort, 64 patients were excluded, 45 due to absence of transcriptomic data and 19 due to non-evaluable pathologic response (e.g., no surgery or ypTX). From the Seiler2017 cohort, 3 patients without information about ypTN status were excluded. The “RC-only” cohort, a Swedish population-based consecutive RC cohort not receiving peri-operative chemotherapy (*n* = 186)^25,37^, was used as comparison to disentangle prognostic from predictive associations.

### Microarray data preprocessing

RNA expression data for the Lund Chemo- and RC-only cohorts were generated on the Affymetrix Human Gene 1.0 ST microarray platform and are available from the Gene Expression Omnibus data repository under accession numbers GSE169455^11^ and GSE83586^37^. Raw data were RMA (Robust Multichip Average) preprocessed and ComBat adjusted to account for differences between labeling batches. Probe sets were then annotated using the BrainArray V25 based on Gencode 36 and summarized at the gene level, resulting in 17,953 unique genes. RNA expression data from Seiler and colleagues^36^ generated on the Affymetrix Human Exon 1.0 ST microarray platform were downloaded from the Gene Expression Omnibus data repository under accession number GSE87304. RNA expression data from the SWOG S1314 trial were generated on the Affymetrix Human Genome U133 Plus 2.0 Array platform and provided by the Southwest Oncology Group. The Seiler2017 and SWOG S1314 datasets were processed in the same way as the Lund cohorts, resulting in 18,869 and 15,987 unique genes, respectively. Genes present in all cohorts were retained for analysis, corresponding to 15,757 genes.

### Lund and consensus subtype classification

Lund molecular subtype classification of all samples was performed using the latest version of the Lund Taxonomy (LundTax) classifier, available through the LundTax2023Classifier R package^31^. Tumors were grouped into the five main molecular subtypes: Urothelial-like (Uro), Genomically unstable (GU), Basal/Squamous (Ba/Sq), Mesenchymal-like (Mes), and Small-cell/Neuroendocrine-like (Sc/Ne). Consensus molecular subtype classification of MIBC was performed using the R package consensusMIBC^29^. Tumors were classified into six molecular subtypes: luminal papillary (LumP), luminal non-specified (LumNS), luminal unstable (LumU), stroma-rich, Ba/Sq, and neuroendocrine-like (Ne-like).

### Meta rank-score calculation and evaluation

In each dataset, differential expression analysis was performed between patients with and without pCR (ypT0N0) using the R package “Limma”. Volcano plots were generated using GraphPad Prism (v10.4.1). For each dataset, genes were ranked from 1, most associated with response, to 15,757, most associated with non-response, based on the moderated t-statistic. To generate a combined ranking across the three NAC-treated cohorts, ranks were weighted by the total number of significant genes in each cohort (adjusted *p* < 0.05), giving greater weight to cohorts with the higher discriminatory power. The weighted rank sum for each gene across the three cohorts was then used to obtain the meta rank-score ranging from 122.3 for the gene most strongly associated with treatment response to 35,336.2 for the gene most strongly associated with non-response. Meta rank-score calculation was performed on the full cohort but also separately for the most prevalent molecular subtype categories; Uro, GU, Ba/Sq, luminal subtypes (Uro or GU), non-luminal subtype (Ba/Sq, Mes, or Sc/Ne), and for treatment modality categories MVAC and GC. Genes were also separately ranked by differential expression analysis in the Lund RC-only cohort. The same analyses were performed using the MIBC consensus classification. Resampling analysis was performed using identical meta rank-scores for 100 permutations in each dataset with random pCR label assignment. The extremes of the permuted meta rank-score distributions were then compared to the observed meta rank-scores. At each position of the distribution, a NAC response signal was defined by a one-sided empirical *p* < 0.05, i.e., if the observed meta rank-score was more extreme than the fifth most extreme permutation.

### GO term analyses, GSEA, PPI analysis and proliferation score analysis

Gene Ontology (GO) enrichment analysis of biological processes was performed using the ShinyGO tool (v0.82)^38^. Terms overrepresented in the 1% or 5% of genes with the highest or lowest meta rank-score were identified for each molecular subtype and treatment category using all common genes as background. Gene set enrichment analysis (GSEA) was performed on the full cohort, and separately for molecular subtype and treatment categories, using GSEA (v4.3.2), scanning H (hallmark), C2 (curated), and C6 (oncogenic) MSigDB gene sets^39^, with genes pre-ranked according to the meta rank-score. Protein-Protein Interaction (PPI) networks were generated using Cytoscape (v3.10.3) and StringApp (v 2.2.0)^40^ on the 1% of genes with the highest or lowest meta rank-score in each subtype. The full STRING network, with a confidence score of 0.7, and a minimum of 5 interactors was used for the analysis. The Proliferation score was calculated using the “LundTaxR” package (https://github.com/mattssca/LundTaxR).

### Immunohistochemical validation

Immunohistochemical (IHC) validation was performed on tissue microarrays (TMA) from the Lund Chemo and RC-only cohorts described previously^11,37^. Briefly, two 1.0 mm tissue cores were obtained per tumor. Cores were placed at representative, tumor-rich areas of the same formalin-fixed paraffin embedded tissue block that was used for RNA extraction. TMA-sections (3 μm) were stained with primary antibodies for SMCO4 (Polyclonal, Proteintech), CYP4Z1 (STJ92604, St John’s Laboratory), CD55 (EPR6689, Abcam), ALDH1A1 (EP1933Y, Abcam), NKG7 (F4V5I, Cell signaling) and CCNB1 (Y106, LifeSpan-BioSciences). Sections were stained on the automated Ventana DISCOVERY ULTRA (Ventana Medical Systems Inc, Tucson, AZ) instrument using DAB for detection, scanned (S60, Hamamatsu), and evaluated as digital images with PathXL Tutor (Cirdan Imaging Limited, UK). Staining was assessed according to the intensity of labeling (score 0–3) and/or the percentage of labeled cells (in 10% increments for cancer cells or stromal cells), depending on each protein’s cellular expression pattern. For proteins positive in both cancer cells and stromal cell types, scores for the two compartments were recorded separately. For each case, the mean value for the two TMA cores was used in the analyses.

### ROC curve and decision curve analysis

Receiver operating characteristic (ROC) curves analyses and decision curve analysis were realized using the “pROC” and “rmda” packages, respectively. For the ROC curves analysis, the area under the curve (AUC) was determined, and 95% confidence intervals (CI) were computed using DeLong’s test. The optimal cutoff value was determined using Youden’s index, defined as the maximum of sensitivity and specificity - 1. At this threshold, sensitivity, specificity, and accuracy were calculated. For decision curves analyses, the net benefit of biomarker-based prediction was compared with default strategies of treating all patients or treating none.

### Statistical analyses

Statistical analyses and visualizations were performed using the R software (v4.3.2) or the GraphPad Prism software (v10.4.1). Fisher’s exact test was used to compare response rates between GU and Ba/Sq in each of the three cohorts. Univariate Cox proportional hazards regression was performed for the Lund Chemo- and RC-only cohorts using the R package “survminer”. Normality tests and two-sided non-parametric tests with a significance threshold of 0.05 were used for validation of biomarkers by IHC.

### Ethics approval and consent to participate

The datasets used in our study contain data that were fully de-identified by the original data providers. No identifiable information was accessible at any stage of our analysis. According to the applicable regulations, ethics approval and informed consent were not required.

## Supplementary information

Supplementary information

Data S1

Data S2

Data S3

## Data Availability

The data generated in this study are available within the article and its supplementary data files. Expression profile data from Lund cohort and Seiler2017 cohort analyzed in this study were obtained from Gene Expression Omnibus (GEO) at GSE169455, GSE83586, and GSE87304. The SWOG S1314 cohort data analyzed in this study were provided by the Southwest Oncology Group. Restrictions apply to the availability of these data, which were used under license for this study.

## References

[1] Advanced Bladder Cancer (ABC) Meta-analysis Collaboration Neoadjuvant chemotherapy in invasive bladder cancer: update of a systematic review and meta-analysis of individual patient data advanced bladder cancer (ABC) meta-analysis collaboration. *Eur. Urol.* **48**, 202–205 (2005). 15939524 10.1016/j.eururo.2005.04.006

[2] Witjes, J. A. et al. European association of urology guidelines on muscle-invasive and metastatic bladder cancer: summary of the 2023 guidelines. *Eur. Urol.* **85**, 17–31 (2024). 37858453 10.1016/j.eururo.2023.08.016

[3] Powles, T. et al. Perioperative durvalumab with neoadjuvant chemotherapy in operable bladder cancer. *N. Engl. J. Med.* **391**, 1773–1786 (2024). 39282910 10.1056/NEJMoa2408154

[4] Vulsteke, C. et al. Perioperative enfortumab vedotin and pembrolizumab in bladder cancer. *N. Engl. J. Med.* **394**, 1257–1269 (2026). 41707170 10.1056/NEJMoa2511674

[5] Rosenblatt, R. et al. Pathologic downstaging is a surrogate marker for efficacy and increased survival following neoadjuvant chemotherapy and radical cystectomy for muscle-invasive urothelial bladder cancer. *Eur. Urol.* **61**, 1229–1238 (2012). 22189383 10.1016/j.eururo.2011.12.010

[6] Lavery, H. J., Stensland, K. D., Niegisch, G., Albers, P. & Droller, M. J. Pathological T0 following radical cystectomy with or without neoadjuvant chemotherapy: a useful surrogate. *J. Urol.* **191**, 898–906 (2014). 24300483 10.1016/j.juro.2013.10.142

[7] Petrelli, F. et al. Correlation of pathologic complete response with survival after neoadjuvant chemotherapy in bladder cancer treated with cystectomy: a meta-analysis. *Eur. Urol.* **65**, 350–357 (2014). 23849998 10.1016/j.eururo.2013.06.049

[8] Bhindi, B. et al. Oncologic outcomes for patients with residual cancer at cystectomy following neoadjuvant chemotherapy: a pathologic stage-matched analysis. *Eur. Urol.* **72**, 660–664 (2017). 28545841 10.1016/j.eururo.2017.05.016

[9] Reesink, D. J., et al. Survival in responders and nonresponders of neoadjuvant and induction chemotherapy in invasive urothelial carcinoma of the urinary bladder: a clinical and pathological stage-matched analysis. *Clin. Genitourin. Cancer* **23**, 102319 (2025). 10.1016/j.clgc.2025.102319 40113474

[10] Taber, A. et al. Molecular correlates of cisplatin-based chemotherapy response in muscle invasive bladder cancer by integrated multi-omics analysis. *Nat. Commun.* **11**, 4858 (2020). 32978382 10.1038/s41467-020-18640-0 PMC7519650

[11] Sjödahl, G. et al. Different responses to neoadjuvant chemotherapy in urothelial carcinoma molecular subtypes. *Eur. Urol.* **81**, 523–532 (2022). 34782206 10.1016/j.eururo.2021.10.035

[12] Groeneveld, C. S. et al. Basal/squamous and mixed subtype bladder cancers present poor outcomes after neoadjuvant chemotherapy in the VESPER trial. *Ann. Oncol.* **36**, 89–98 (2025). 39299443 10.1016/j.annonc.2024.09.008

[13] Börcsök, J. et al. Quantitative functional profiling of ERCC2 mutations deciphers cisplatin sensitivity in bladder cancer. *J. Clin. Invest.* **135**, e186688 (2025). 40829180 10.1172/JCI186688 PMC12352908

[14] Vollmer, T. et al. The intratumoral CXCR3 chemokine system is predictive of chemotherapy response in human bladder cancer. *Sci. Transl. Med.* **13**, eabb3735 (2021). 33441425 10.1126/scitranslmed.abb3735

[15] Nassif, E. F. et al. The immunoscore in localized urothelial carcinoma treated with neoadjuvant chemotherapy: clinical significance for pathologic responses and overall survival. *Cancers* **13**, 494 (2021). 33525361 10.3390/cancers13030494 PMC7865364

[16] Choi, W. et al. Identification of distinct basal and luminal subtypes of muscle-invasive bladder cancer with different sensitivities to frontline chemotherapy. *Cancer Cell* **25**, 152–165 (2014). 24525232 10.1016/j.ccr.2014.01.009 PMC4011497

[17] Mi, H. et al. Predictive models of response to neoadjuvant chemotherapy in muscle-invasive bladder cancer using nuclear morphology and tissue architecture. *Cell Rep. Med.* **2**, 100382 (2021). 34622225 10.1016/j.xcrm.2021.100382 PMC8484511

[18] Liu, D. et al. Clinical validation of chemotherapy response biomarker ERCC2 in muscle-invasive urothelial bladder carcinoma. *JAMA Oncol.* **2**, 1094–1096 (2016). 27310333 10.1001/jamaoncol.2016.1056 PMC5515075

[19] Gil-Jimenez, A. et al. Assessment of predictive genomic biomarkers for response to cisplatin-based neoadjuvant chemotherapy in bladder cancer. *Eur. Urol.* **83**, 313–317 (2023). 35965206 10.1016/j.eururo.2022.07.023

[20] Khurana, S. & George, S. P. Regulation of cell structure and function by actin-binding proteins: villin’s perspective. *FEBS Lett.* **582**, 2128–2139 (2008). 18307996 10.1016/j.febslet.2008.02.040 PMC2680319

[21] Fleischmann, A., Thalmann, G. N., Perren, A. & Seiler, R. Tumor regression grade of urothelial bladder cancer after neoadjuvant chemotherapy: a novel and successful strategy to predict survival. *Am. J. Surg. Pathol.* **38**, 325–332 (2014). 24525502 10.1097/PAS.0000000000000142

[22] Beckabir, W. et al. Spatial relationships in the tumor microenvironment demonstrate association with pathologic response to neoadjuvant chemoimmunotherapy in muscle-invasive bladder cancer. *Eur. Urol.* **85**, 242–253 (2024). 38092611 10.1016/j.eururo.2023.11.008 PMC11022933

[23] Baras, A. S. et al. The ratio of CD8 to Treg tumor-infiltrating lymphocytes is associated with response to cisplatin-based neoadjuvant chemotherapy in patients with muscle invasive urothelial carcinoma of the bladder. *Oncoimmunology* **5**, e1134412 (2016). 27467953 10.1080/2162402X.2015.1134412 PMC4910705

[24] Galsky, M. D. et al. Immunomodulatory effects and improved outcomes with cisplatin- versus carboplatin-based chemotherapy plus atezolizumab in urothelial cancer. *Cell Rep. Med.* **5**, 101393 (2024). Feb 20. 38280376 10.1016/j.xcrm.2024.101393 PMC10897541

[25] Kollberg, P., Chebil, G., Eriksson, P., Sjödahl, G. & Liedberg, F. Molecular subtypes applied to a population-based modern cystectomy series do not predict cancer-specific survival. *Urol. Oncol.* **37**, 791–799 (2019). 31056435 10.1016/j.urolonc.2019.04.010

[26] Stein, J. P. et al. Radical cystectomy in the treatment of invasive bladder cancer: long-term results in 1,054 patients. *J. Clin. Oncol.* **41**, 3772–3781 (2023). 37499357 10.1200/JCO.22.02762

[27] Brant, A. et al. Pathologic response in patients receiving neoadjuvant chemotherapy for muscle-invasive bladder cancer: is therapeutic effect owing to chemotherapy or TURBT? *Urol. Oncol.* **35**, 34.e17–34.e25 (2017). 27639777 10.1016/j.urolonc.2016.08.005

[28] de Angelis, M. et al. Neoadjuvant chemotherapy before radical cystectomy in patients with organ-confined and non-organ-confined urothelial carcinoma. *Urol. Oncol.* **43**, 62.e1–62.e6 (2025). 39424431 10.1016/j.urolonc.2024.09.015

[29] Kamoun, A. et al. A consensus molecular classification of muscle-invasive bladder cancer. *Eur. Urol.* **77**, 420–433 (2020). 31563503 10.1016/j.eururo.2019.09.006 PMC7690647

[30] Höglund, M. What is a bladder cancer molecular subtype? *Bladder Cancer* **9**, 293–298 (2023). 38994241 10.3233/BLC-220124 PMC11165936

[31] Cotillas, E. A. et al. A versatile and upgraded version of the LundTax classification algorithm applied to independent cohorts. *J. Mol. Diagn.* **26**, 1081–1101 (2024). 39326668 10.1016/j.jmoldx.2024.08.005

[32] Murphy, N. et al. Predictive molecular biomarkers for determining neoadjuvant chemosensitivity in muscle invasive bladder cancer. *Oncotarget* **13**, 1188–1200 (2022). 36322407 10.18632/oncotarget.28302 PMC9629806

[33] Conroy, S. et al. Ex vivo drug screening and clustering of bladder cancers for pre-clinical treatment prediction. *Commun. Med*. (2026). 10.1038/s43856-026-01596-5 PMC13408482 42135441

[34] Flaig, T. W. et al. A randomized phase II study of coexpression extrapolation (COXEN) with neoadjuvant chemotherapy for bladder cancer (SWOG S1314; NCT02177695). *Clin. Cancer Res.* **27**, 2435–2441 (2021). 33568346 10.1158/1078-0432.CCR-20-2409 PMC8219246

[35] Lerner, S. P. et al. Association of molecular subtypes with pathologic response, PFS, and OS in a phase II study of COXEN with neoadjuvant chemotherapy for muscle-invasive bladder cancer. *Clin. Cancer Res* **30**, 444–449 (2024). 37966367 10.1158/1078-0432.CCR-23-0602 PMC10824507

[36] Seiler, R. et al. Impact of molecular subtypes in muscle-invasive bladder cancer on predicting response and survival after neoadjuvant chemotherapy. *Eur. Urol.* **72**, 544–554 (2017). 28390739 10.1016/j.eururo.2017.03.030

[37] Sjödahl, G., Eriksson, P., Liedberg, F. & Höglund, M. Molecular classification of urothelial carcinoma: global mRNA classification versus tumour-cell phenotype classification. *J. Pathol.* **242**, 113–125 (2017). 28195647 10.1002/path.4886 PMC5413843

[38] Ge, S. X., Jung, D. & Yao, R. ShinyGO: a graphical gene-set enrichment tool for animals and plants. *Bioinformatics* **36**, 2628–2629 (2020). 31882993 10.1093/bioinformatics/btz931 PMC7178415

[39] Subramanian, A. et al. Gene set enrichment analysis: a knowledge-based approach for interpreting genome-wide expression profiles. *Proc. Natl. Acad. Sci. USA.* **102**, 15545–15550 (2005). 16199517 10.1073/pnas.0506580102 PMC1239896

[40] Doncheva, N. T., Morris, J. H., Gorodkin, J. & Jensen, L. J. Cytoscape StringApp: network analysis and visualization of proteomics data. *J. Proteome Res.* **18**, 623–632 (2019). 30450911 10.1021/acs.jproteome.8b00702 PMC6800166

