# Supplementary material for: Proliferation- and cytotoxic immune signatures identify chemotherapy-responsive bladder tumors in a molecular subtype dependent manner
Source: NPJ Precis Oncol. 2026 Jul 3;10:258. doi: 10.1038/s41698-026-01588-7 (PMC13332042; doi:10.1038/s41698-026-01588-7)
Supplement: Supplementary file 1 — Supplementary information [file 41698_2026_1588_MOESM1_ESM.pdf]

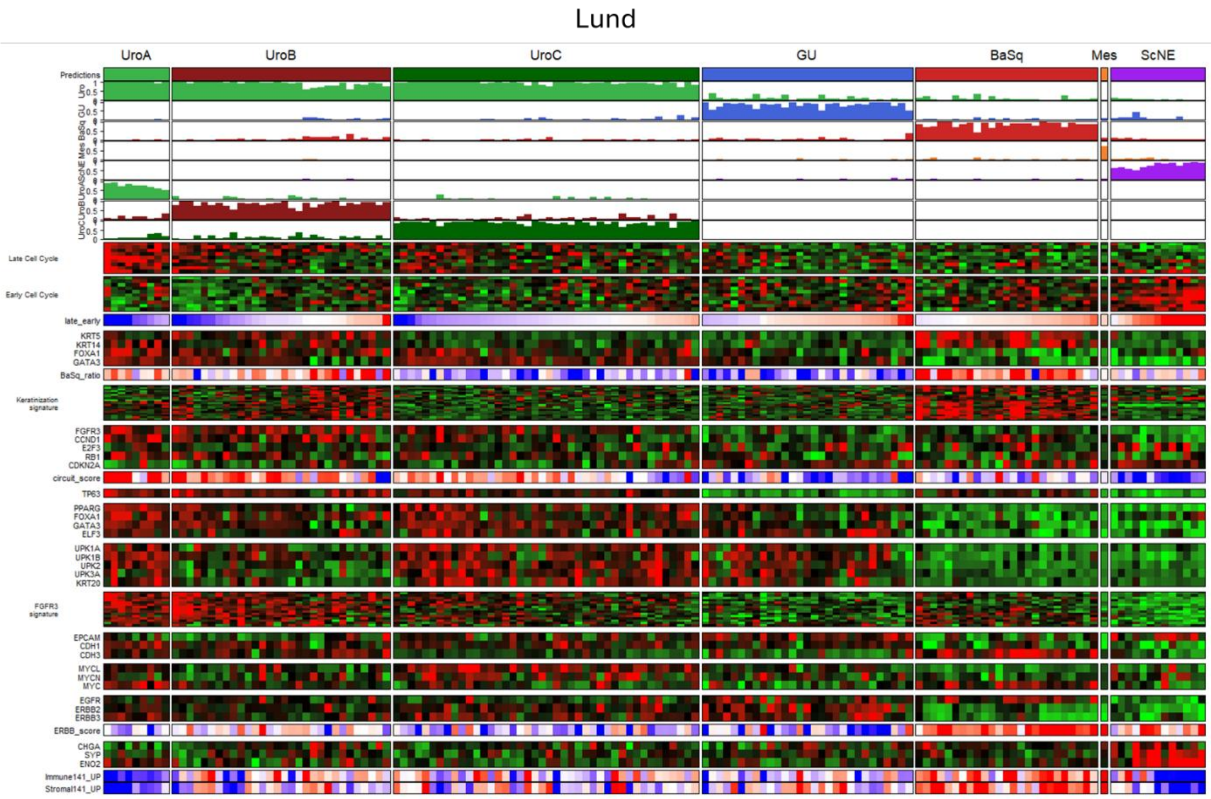

Figure S1. Heatmap showing the subtype classification of the Lund NAC cohort

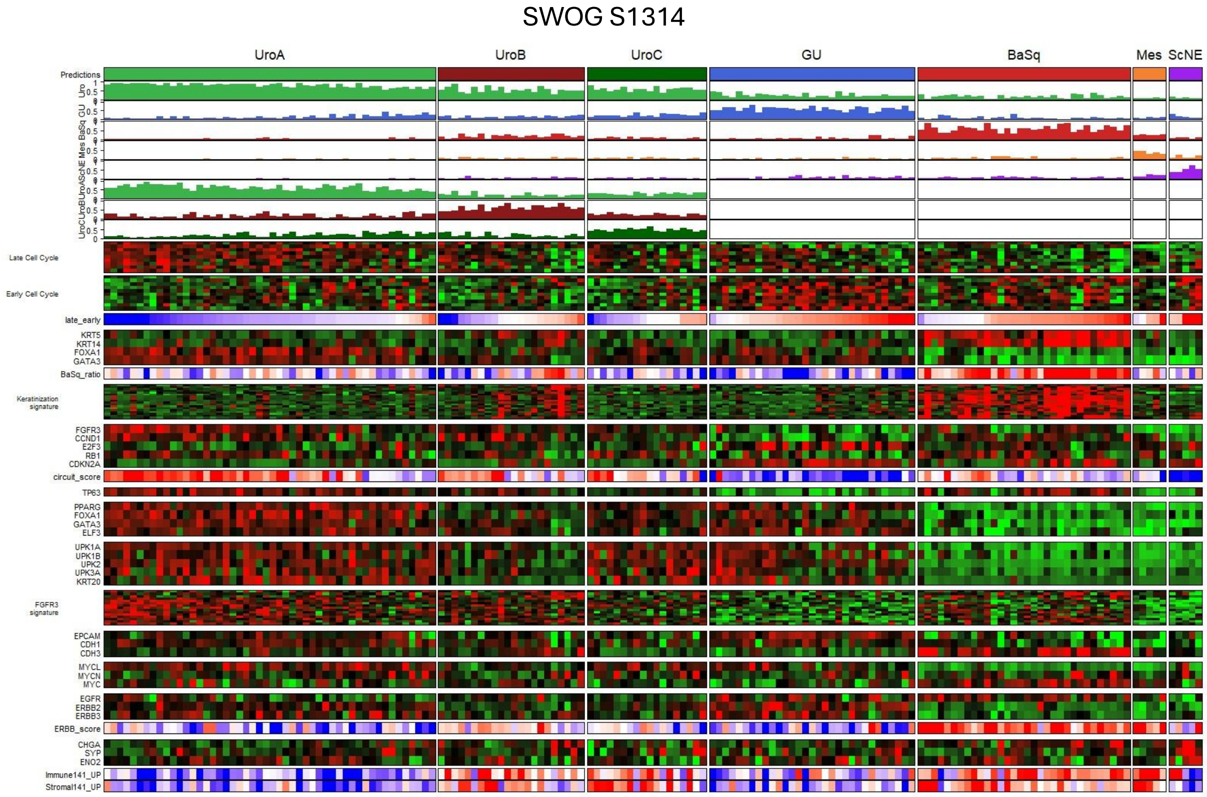

Figure S2. Heatmap showing the subtype classification of the SWOG S1314 cohort

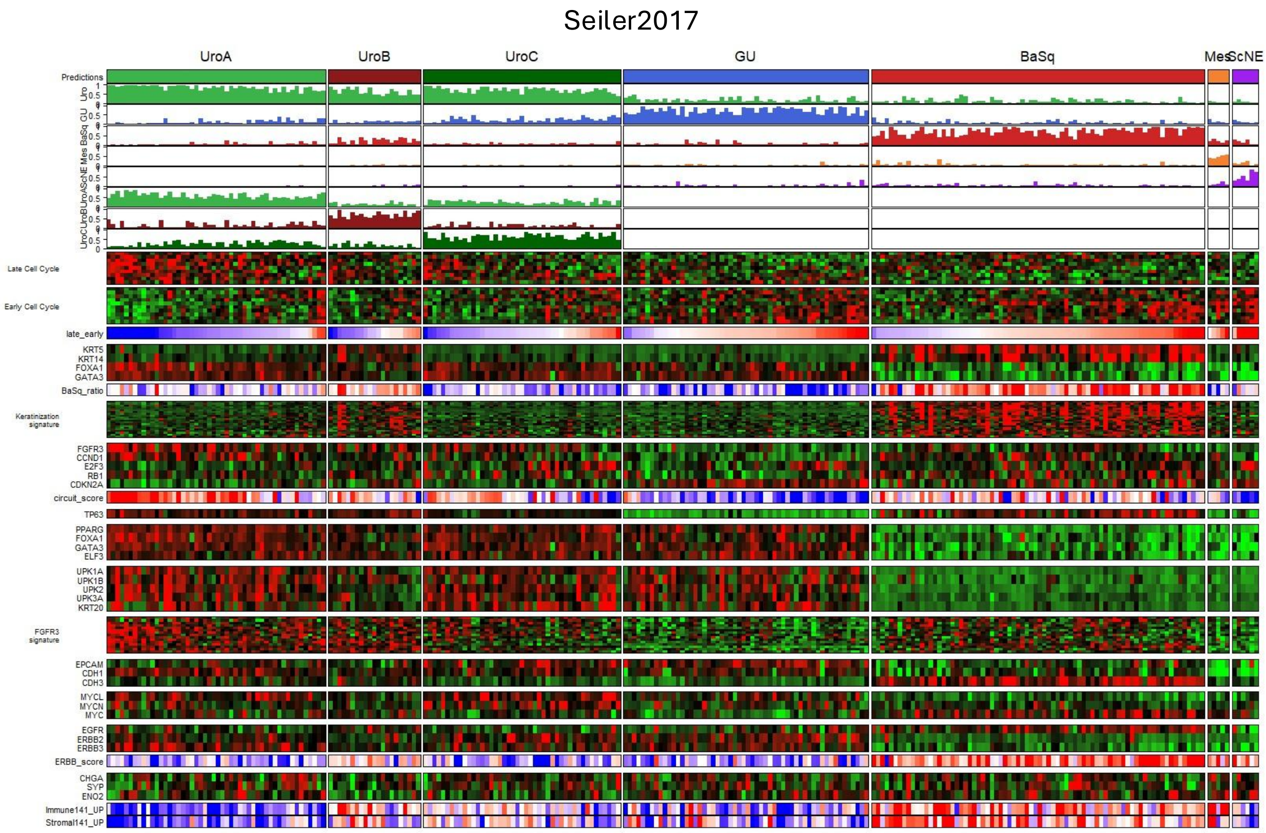

Figure S3. Heatmap showing the subtype classification of the Seiler2017 cohort

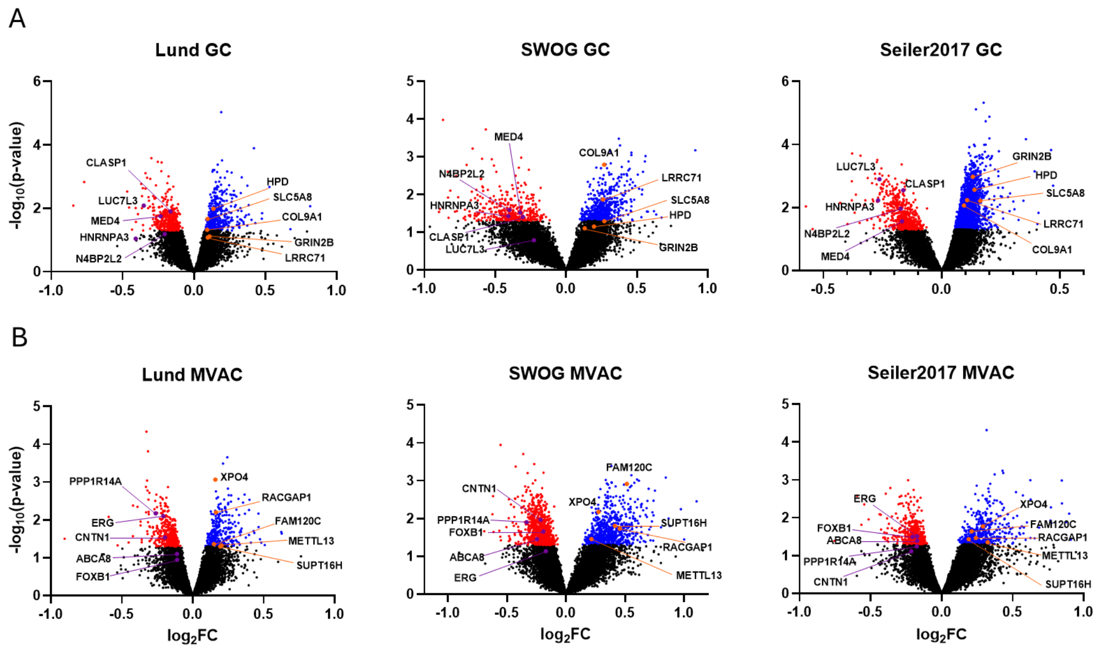

Figure S4. Differential expression analysis by treatment response in each cohort stratified by treatment regimen. *A-B. Volcano plots representing the differential expression analyses performed on the MVAC-treated subgroup (A) and GC-treated subgroup (B). Genes with statistical significance (p≤0.05) are marked in red for downregulation and blue for upregulation, and the 5 genes with the highest and lowest meta rank-score in each volcano plot are highlighted.*

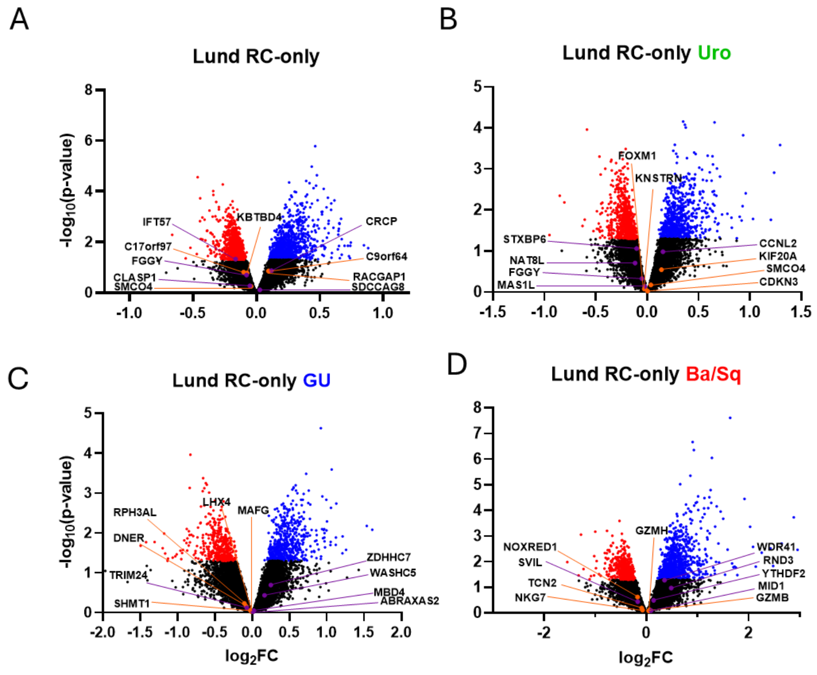

Figure S5. Differential expression analysis by treatment response in the full Lund RC-only cohort and in three molecular subtypes. *A-D. Volcano plots showing differentially expressed genes the full Lund RC-only cohort (A) and in the Uro (B), GU (C) and Ba/Sq (D) subsets. Statistically significant genes are marked in red for downregulation and blue for upregulation, and the 5 genes with the highest and lowest meta rank-score in each volcano plot are highlighted.*

Data S1. Meta rank-score tables. *Differential expression analysis was performed and used to compute a meta rank-score. Genes were ranked according to this score, from lowest to highest. Analyses were performed in the full cohort and in molecular subtypes according to the Lund taxonomy.*

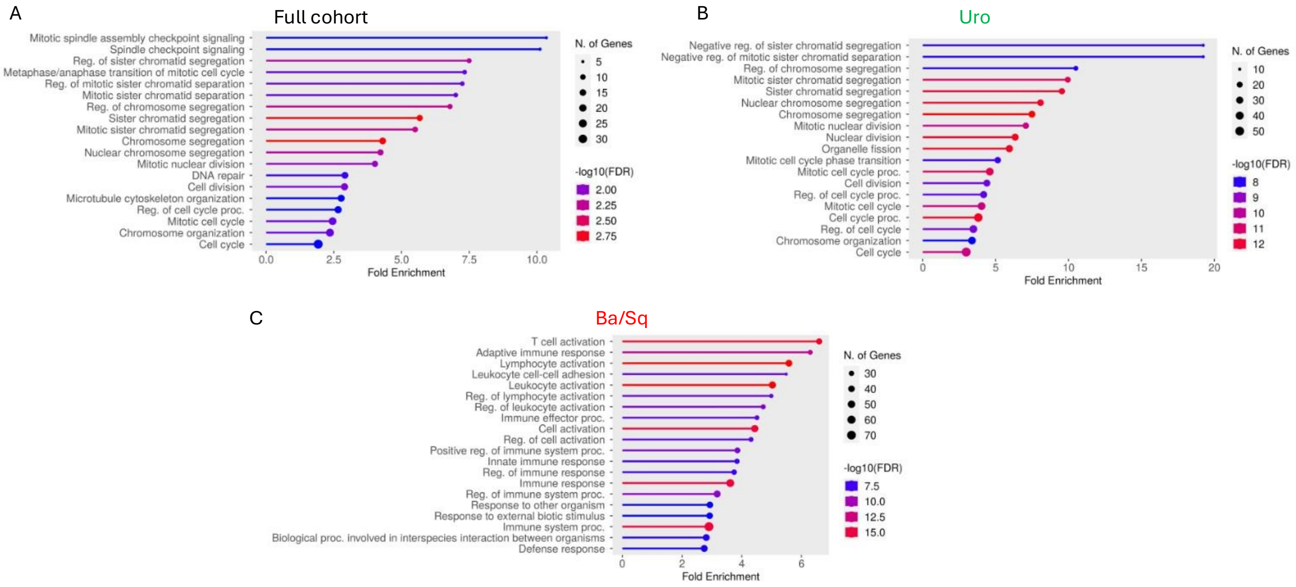

Figure S6. Top 20 subtype-specific, response associated GO terms. *GO term analysis using 'biological processes', for the 1% of genes with the lowest meta rank-score in the full cohort (A) and major molecular subtypes: Uro (B) and Ba/Sq (C). GU subtype, which had no significantly enriched processes in the top 1% of genes, is not shown.*

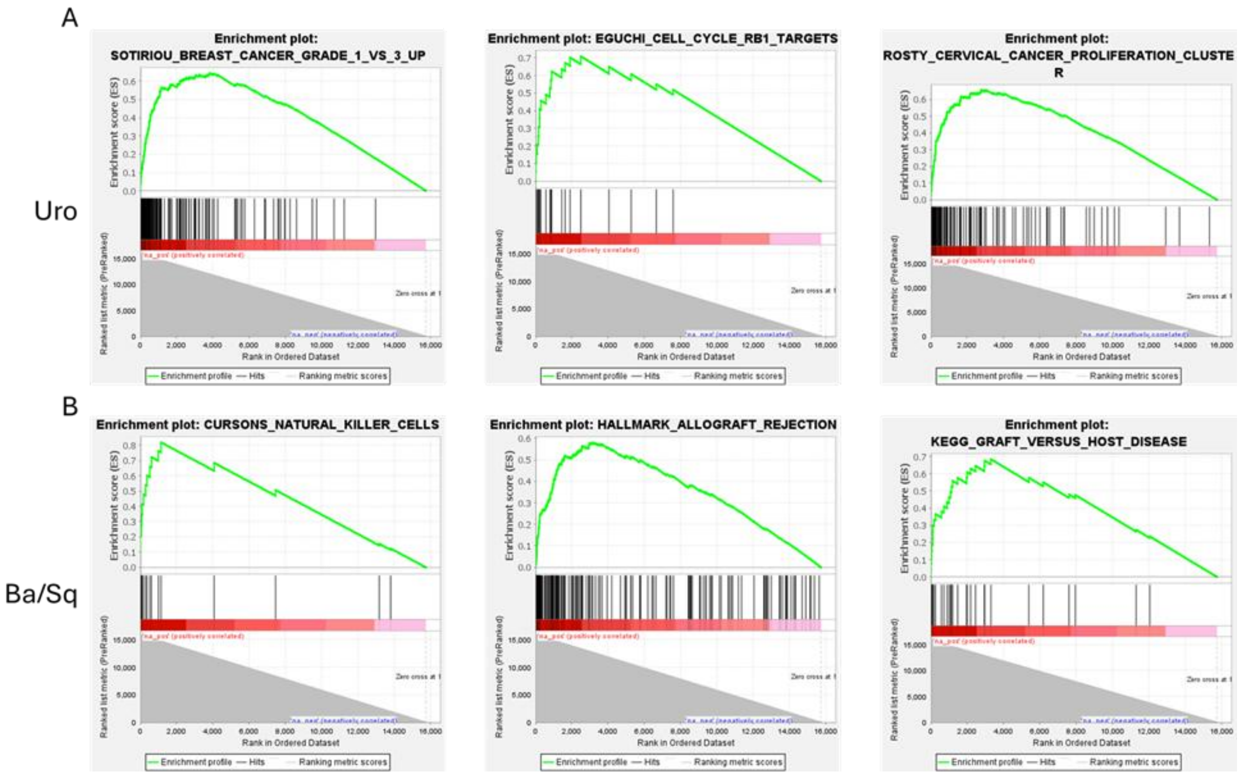

Figure S7. The GSEA highlighted enrichments specific to molecular subtype. *A and B: GSEA analysis confirmed robust enrichment of cell cycle-related signatures in the Uro subtype (A), and cytotoxic immune-related signatures in the Ba/Sq subtype (B), both positively associated with pathologic response.*

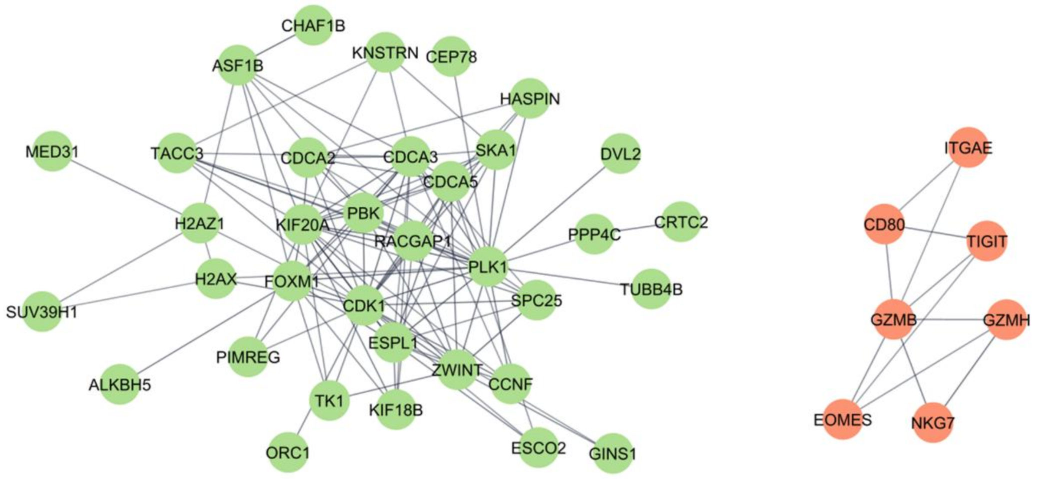

Figure S8. The Uro subtype drives the protein interactome in the full cohort. *Major protein-protein interaction observed in the top 1% of genes with the lowest meta rank-score in the full cohort using STRING. The proliferation module enriched in the Uro subtype is identified, as well as a limited cytotoxic immune module enriched in Ba/Sq.*

Data S2. Meta rank-score tables for the consensus classification. *Differential expression analysis was performed and used to compute a meta rank-score. Genes were ranked according to this score, from lowest to highest. Analyses were performed in the full cohort and in molecular subtypes according to the consensus classification*

A

Lund cohort

Lund\consensus	LumP	LumNS	LumU	Ba/Sq	Stroma-rich	NE-like	Total
Uro	39	18	7	7	10	0	81
GU	1	2	12	2	12	0	29
Ba/Sq	0	0	0	22	3	0	25
ScNE	0	0	0	0	4	9	13
Mes	0	0	0	0	1	0	1
Total	40	20	19	31	30	9	149

Lund\consensus	LumP	LumNS	LumU	Ba/Sq	Stroma-rich	NE-like	Total
UroA	9	0	0	0	0	0	9
UroB	18	1	0	7	4	0	30
UroC	12	17	7	0	6	0	42
GU	1	2	12	2	12	0	29
Ba/Sq	0	0	0	22	3	0	25
ScNE	0	0	0	0	4	9	13
Mes	0	0	0	0	1	0	1
Total	40	20	19	31	30	9	149

B

Seiler2017

Lund\consensus	LumP	LumNS	LumU	Ba/Sq	Stroma-rich	NE-like	Total
Uro	44	34	17	9	11	0	115
GU	3	6	32	1	15	0	57
Ba/Sq	0	0	0	67	9	0	76
ScNE	0	0	0	3	0	3	6
Mes	0	0	0	4	1	0	5
Total	47	40	49	84	36	3	259

Lund\consensus	LumP	LumNS	LumU	Ba/Sq	Stroma-rich	NE-like	Total
UroA	32	14	2	0	2	0	50
UroB	8	1	0	9	3	0	21
UroC	4	19	15	0	6	0	44
GU	3	6	32	1	15	0	57
Ba/Sq	0	0	0	67	9	0	76
ScNE	0	0	0	3	0	3	6
Mes	0	0	0	4	1	0	5
Total	47	40	49	84	36	3	259

C

SWOG S1314

Lund\Consensus	LumP	LumNS	LumU	Ba/Sq	Stroma-rich	NE-like	Total
Uro	45	21	5	9	10	0	90
GU	3	2	17	4	5	0	31
Ba/Sq	0	0	0	32	0	0	32
ScNE	0	0	0	3	0	2	5
Mes	0	0	0	4	1	0	5
Total	48	23	22	52	16	2	163

Lund\consensus	LumP	LumNS	LumU	Ba/Sq	Stroma-rich	NE-like	Total
UroA	39	9	2	0	0	0	50
UroB	4	4	0	9	5	0	22
UroC	2	8	3	0	5	0	18
GU	3	2	17	4	5	0	31
Ba/Sq	0	0	0	32	0	0	32
ScNE	0	0	0	3	0	2	5
Mes	0	0	0	4	1	0	5
Total	48	23	22	52	16	2	163

Figure S9. Concordance between the LundTax and the consensus classifier. *The closest subtypes between the LundTax and consensus classifications are highlighted in green.*

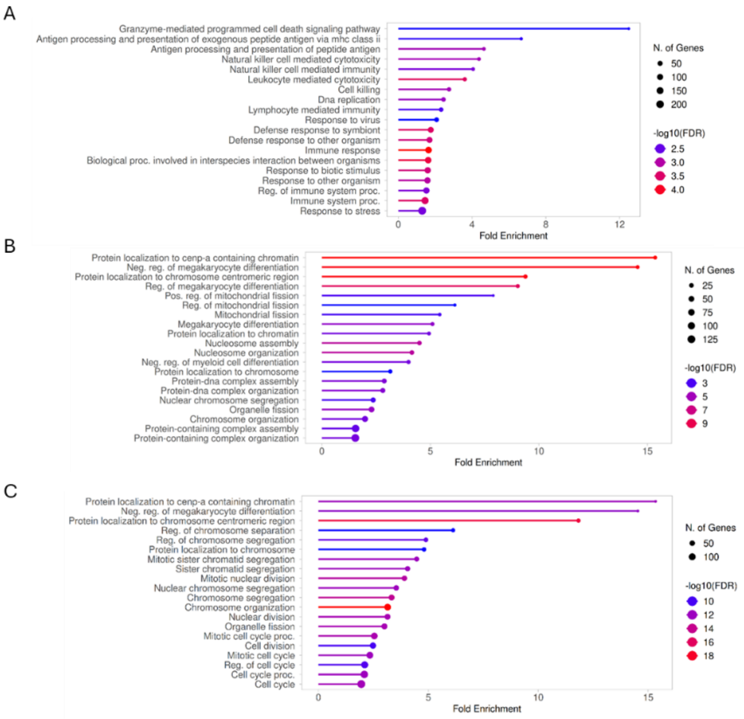

Figure S10. Top 20 consensus subtype-specific, response associated GO terms. *GO term analysis using 'biological processes', for the 5% of genes with the lowest meta rank-score in the consensus subtypes: Ba/Sq (A), LumNS (B) and the combination of LumNS and LumP (C). Stroma rich and LumU showed no enriched processes in the top 1% of genes.*

Data S3. Pairwise overlap of top differentially expressed genes in the three NAC cohorts. The meta rank-score table was used to determine the number of overlapping genes across the different cohorts.

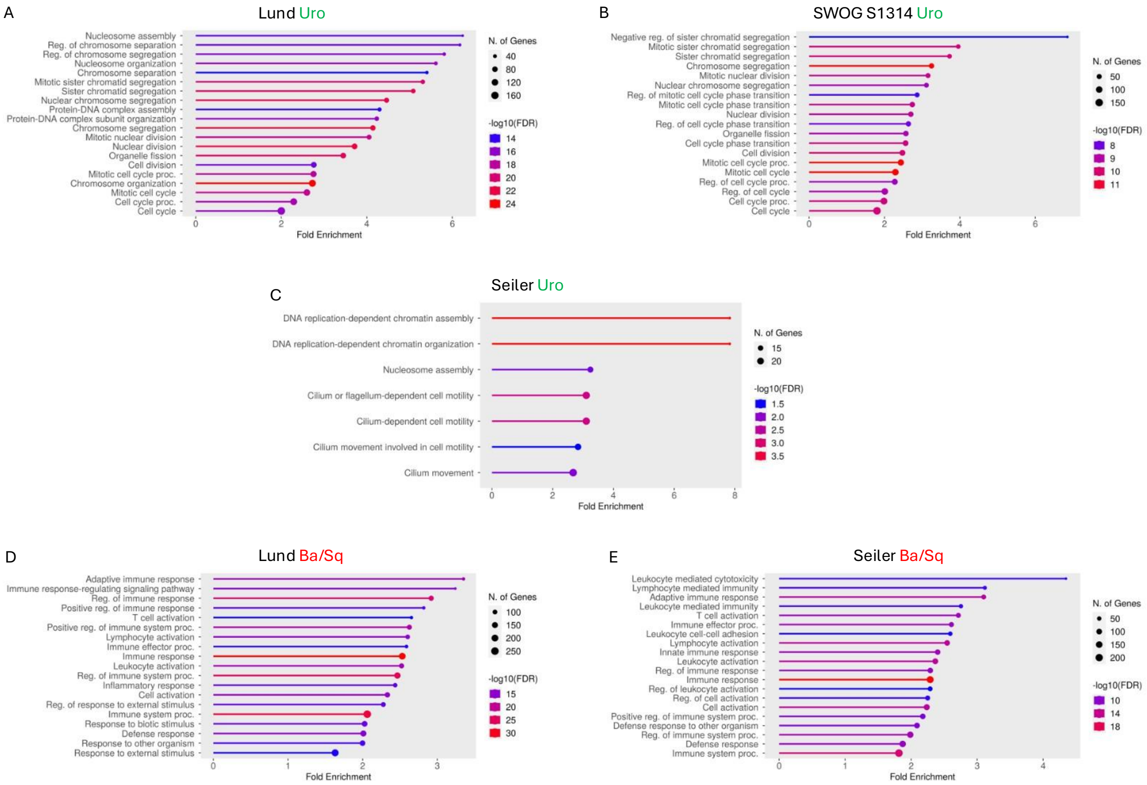

Figure S11. Enrichment analyses are improved by combining the three cohorts. *GO term analysis using 'biological processes', for the 5% most response-associated genes in the Uro and Ba/Sq subsets in each cohort. The Ba/Sq subtype in the SWOG S1314 cohort showed no significantly enriched processes.*

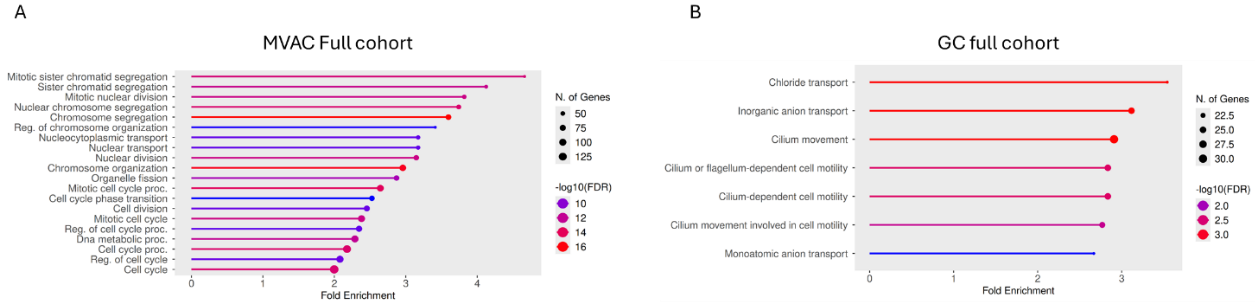

Figure S12. Proliferation-related pathway enrichment is predominantly observed in patients treated with MVAC. *GO term analysis using 'biological processes', for the 5% genes with the lowest meta rank-score. Analyses were conducted in the full cohort stratified by treatment regimen: MVAC (A) vs GC (B). Proliferation signatures were enriched in responders among patients treated with MVAC (A) but not among those treated with GC (B).*

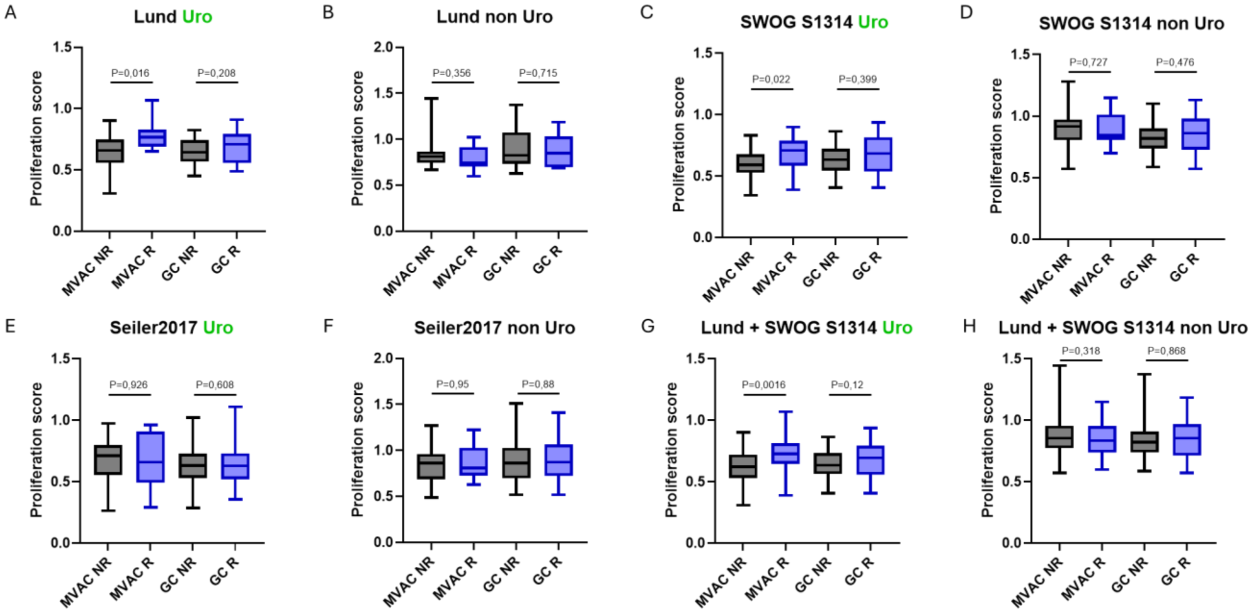

Figure S13. Proliferation score is associated with response in the Uro subtype regardless of the treatment regimen. *A-H: Proliferation scores were computed for each patient and compared based on treatment response and treatment regimen within the Uro and non-Uro subtypes for the Lund cohort (A and B), SWOG S1314 (C and D), Seiler 2017 (E and F) and the combination of Lund and SWOG S1314 (G and H). In the Lund and SWOG S1314 cohorts, the association between proliferation and response was limited to the Uro subtype, within which it was statistically significant only in the MVAC treated subset (A-D). In the Seiler cohort, proliferation was not associated with response in any subset (E-F). When combining the Lund and SWOG S1314 cohorts, proliferation was associated with a better response only within the Uro subtype, with a stronger effect in the MVAC treated Uro tumors than in the GC treated Uro tumors (G). Notably, in non-Uro subtypes, proliferation showed no association with response in either regimen (H).*

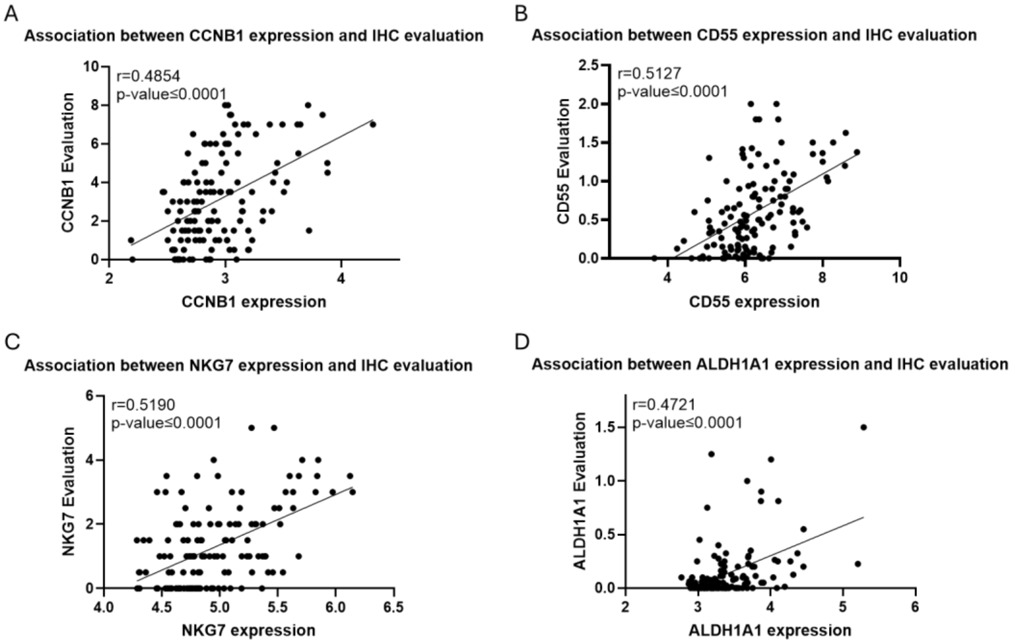

Figure S14. The IHC evaluation of different biomarkers correlates with their gene expression. *Scatter plots showing a significant positive correlation (p ≤ 0.0001) between IHC evaluations from TMAs and matched mRNA expression for CCNB1 (A), CD55 (B), NKG7 (C), and ALDH1A1 (D) in the Lund NAC cohort.*

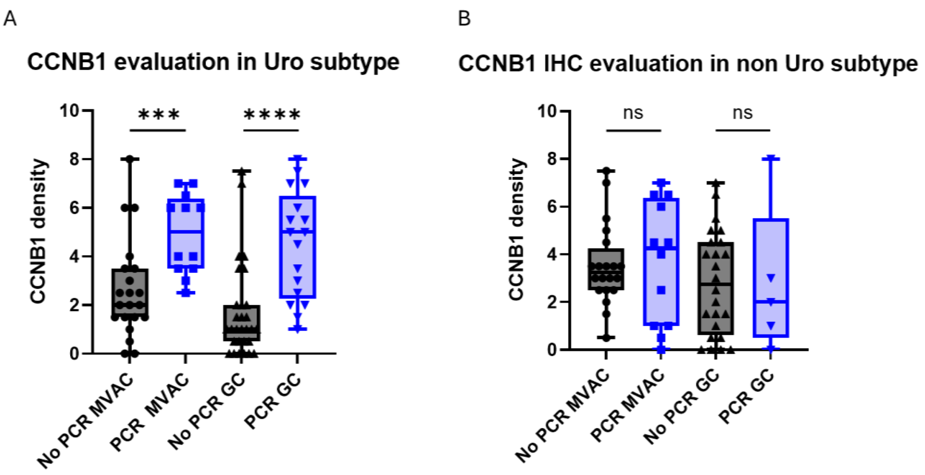

Figure S15. The association between IHC evaluation of CCNB1 and response in Uro subtype is independent of the treatment regimen. *A-B: CCNB1 expression was evaluated in cancer cells. Expression levels were then compared across tumor subtypes stratified by both treatment response and treatment regimen: Uro tumors treated with MVAC or GC (A) and non Uro tumors treated with MVAC or GC (B).*

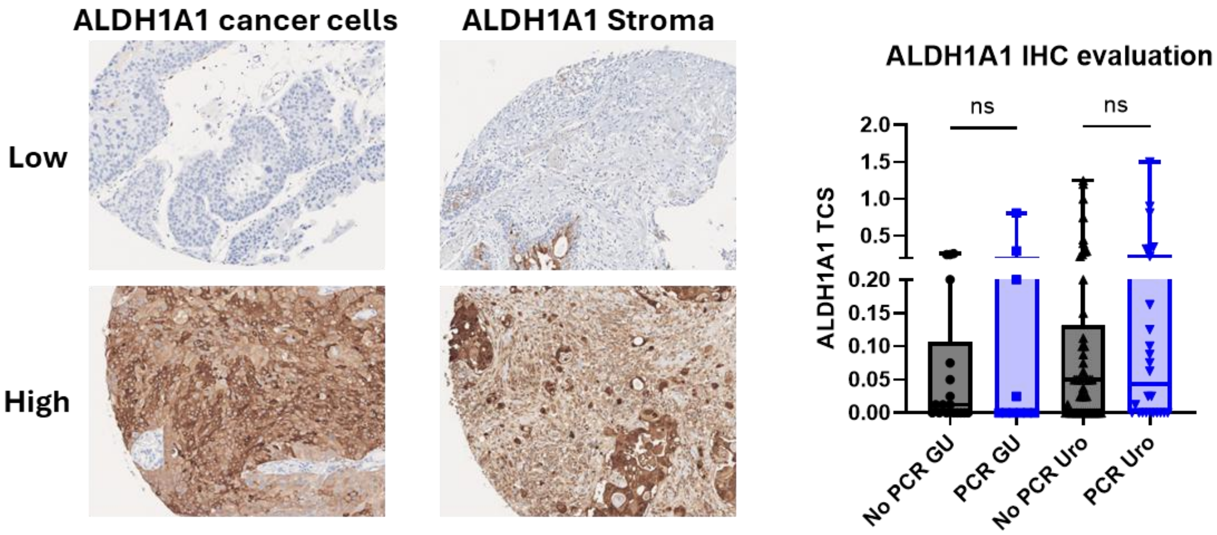

Figure S16. IHC evaluation of ALDH1A1 does not confirm its potential role as a biomarker. *The upper panels show examples of absent/weak ALDH1A1 staining in the cancer cells and in the stroma. The lower panels show examples with the strongest staining in the cancer cells and in the stroma. Boxplots show combined staining scores in the Uro and GU subtypes stratified by pathologic response.*

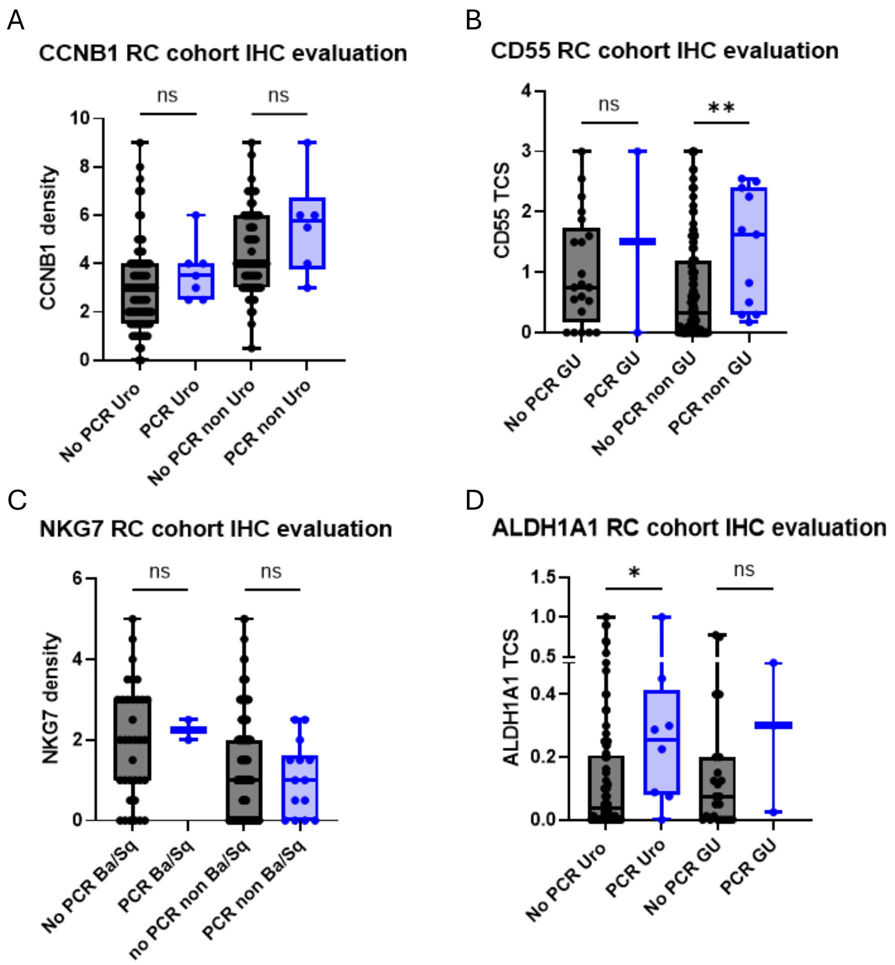

Figure S17. NKG7 and CCNB1 serve as biomarkers of therapeutic response, but not as prognostic markers. Density of *CCNB1 positive cancer cells, density of NKG7 positive immune cells, and quantitative expression in both cancer cell and stroma compartments for CD55 and ALH1A1 is shown in the LUND RC-only cohort. Analyses are stratified by each marker's subtype-specific association patterns and by pathologic response: Uro versus non-Uro for CCNB1 (A), GU versus non-GU for CD55 (B), Ba/Sq versus non-Ba/Sq for NKG7 (C) and Uro versus GU for ALDH1A1 (D).*

A

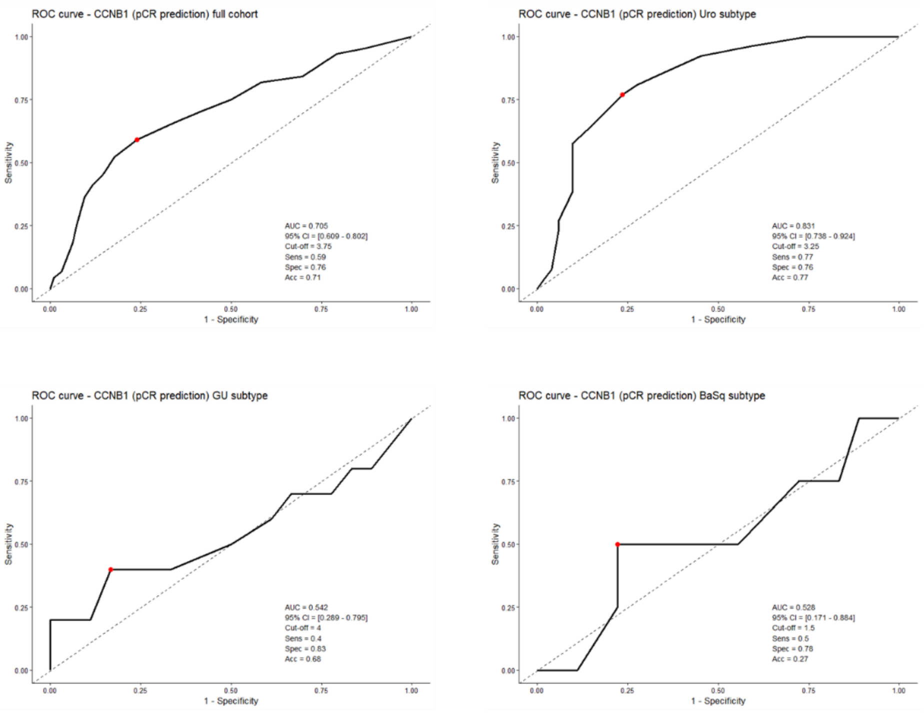

B

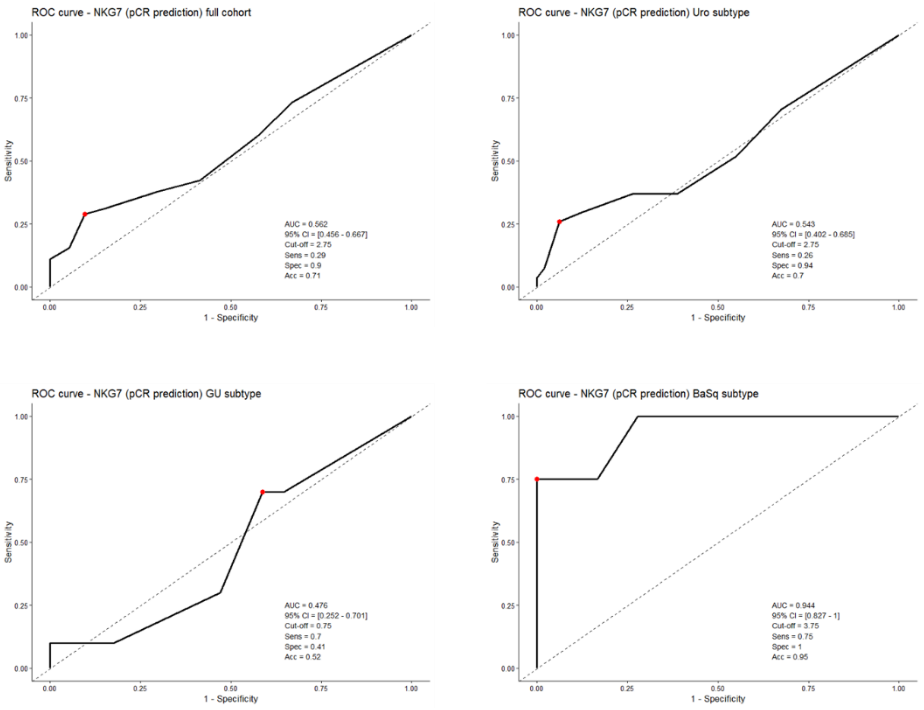

Figure S18. IHC evaluation of CCNB1 and NKG7 predict PCR in the Lund cohort in a subtype-specific manner. *A and B: ROC curves analysis showing the predictive performance of IHC evaluation of CCNB1 (A) and NKG7 (B) for pCR in the Lund cohort were performed in the full cohort as well as in the Uro, Ba/Sq, and GU subtypes. The AUC and corresponding 95% CI, cut-off value, sensitivity, specificity, and accuracy are shown.*

A

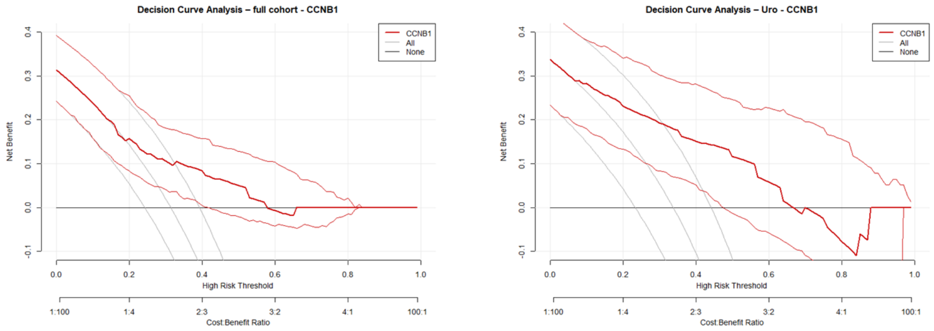

B

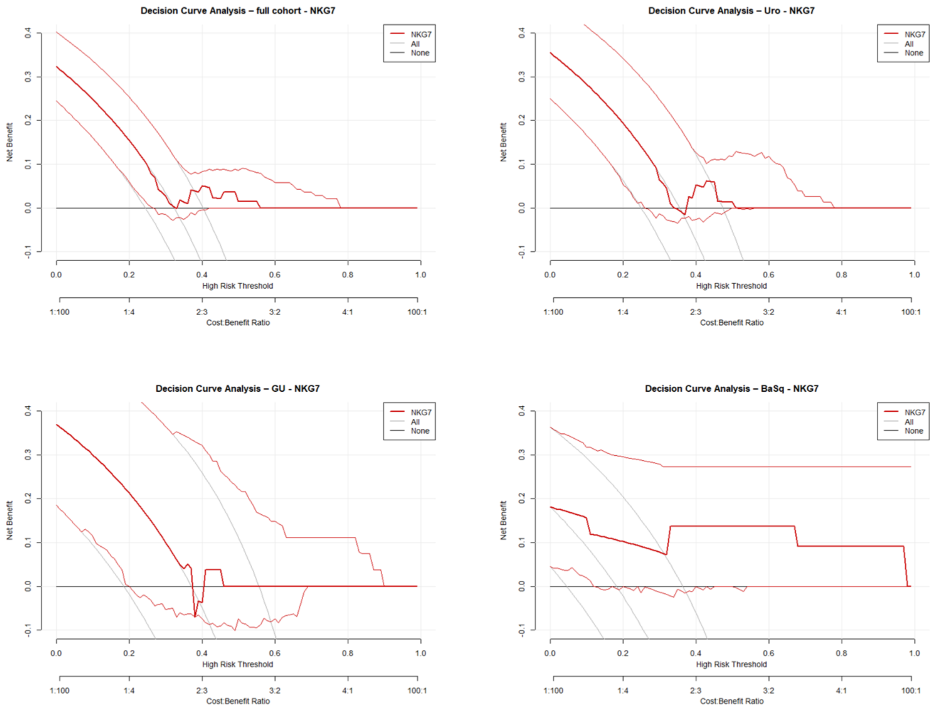

Figure S19. Decision curve analysis of IHC evaluation of CCNB1 and NKG7 for prediction of pCR. *A and B: Decision curve analysis of IHC evaluation of CCNB1 and NKG7 for pCR were performed in the Lund cohort in the full cohort as well as in the Uro, Ba/Sq, and GU subtypes.*
